# Cargo receptor Surf4 regulates endoplasmic reticulum export of proinsulin in pancreatic β-cells

**DOI:** 10.1038/s42003-022-03417-6

**Published:** 2022-05-13

**Authors:** Keiko Saegusa, Kohichi Matsunaga, Miharu Maeda, Kota Saito, Tetsuro Izumi, Ken Sato

**Affiliations:** 1 Laboratory of Molecular Traffic, Institute for Molecular and Cellular Regulation, Gunma University, Maebashi, Gunma, 371-8512 Japan; 2 Laboratory of Molecular Endocrinology and Metabolism, Department of Molecular Medicine, Institute for Molecular and Cellular Regulation, Gunma University, Maebashi, Gunma, 371-8512 Japan; 3 Department of Biological Informatics and Experimental Therapeutics, Graduate School of Medicine, Akita University, Akita, 010-8543 Japan; 4 Gunma University, Initiative for Advanced Research (GIAR), Gunma, 371-8512 Japan

**Keywords:** Endoplasmic reticulum, Endoplasmic reticulum

## Abstract

Insulin is an essential peptide hormone that maintains blood glucose levels. Although the mechanisms underlying insulin exocytosis have been investigated, the mechanism of proinsulin export from the endoplasmic reticulum (ER) remains unclear. Here, we demonstrated that Surf4, a cargo receptor homolog, regulates the ER export of proinsulin via its recruitment to ER exit sites (ERES). Under high-glucose conditions, Surf4 expression was upregulated, and Surf4 proteins mainly localized to the ER at a steady state and accumulated in the ERES, along with proinsulin in rat insulinoma INS-1 cells. *Surf4*-knockdown resulted in proinsulin retention in the ER and decreased the levels of mature insulin in secretory granules, thereby significantly reducing insulin secretion. Surf4 forms an oligomer and can physically interact with proinsulin and Sec12, essential for COPII vesicle formation. Our findings suggest that Surf4 interacts with proinsulin and delivers it into COPII vesicles for ER export in co-operation with Sec12 and COPII.

## Abstract (web summary)

Surf4 is an ER cargo receptor for proinsulin, facilitating proinsulin export out of the ERES in a cellular model of pancreatic β-cells, with its expression dependent on glucose concentration and Surf4 knockdown impairing insulin secretion.

## Introduction

Insulin, a hormone secreted by pancreatic β cells, lowers blood glucose concentration by promoting the uptake of circulating glucose into various tissues, such as skeletal muscles, and adipose tissues. Insulin secretion is enhanced by postprandial hyperglycemia, and its impairment causes prolonged high levels of blood glucose, often accompanied by the onset and progression of type 2 diabetes^1–3^.

Insulin release is carried out by the exocytotic machinery that regulates the fusion of insulin-containing secretory granules (insulin granules) with the plasma membrane in response to intracellular metabolic signals^4^. Thus, to sustain insulin release during prolonged secretion, β cells need to accelerate the *de novo* synthesis of insulin at the endoplasmic reticulum (ER) and transport them to the trans-Golgi network (TGN) where insulin granules are generated.

Insulin is translated as a precursor called preproinsulin that is translocated into the ER lumen upon the cleavage of its signal peptide to produce proinsulin. Proinsulin is then folded correctly by ER chaperones and protein disulfide isomerase (PDI) to form a homo-oligomer^5–8^. Properly folded proinsulin is transported from the ER to the Golgi apparatus and packaged into specialized secretory granules called as insulin granules at TGN. Proinsulin is then gradually converted to its bioactive mature insulin by prohormone convertases PC1/3 and PC2 and carboxypeptidase E as insulin granules mature^9,10^.

Recent studies have revealed that defective ER export impairs proinsulin oxidative folding and induces ER stress in β cells, suggesting that efficient ER export of proinsulin is essential for ER homeostasis and prevents β cell dysfunction^11–13^. Moreover, the downregulation of Sar1A, a key regulator of coat protein complex II (COPII)-coated vesicle formation, inhibited the ER export of proinsulin, indicating that the transport of proinsulin from the ER into the Golgi apparatus is mediated by COPII-coated transport vesicles^12^. However, although the mechanism of exocytosis of insulin granules has been well studied, the mechanistic basis for the sorting and packaging of proinsulin into COPII-coated vesicles remains poorly understood.

ER–Golgi trafficking of secretory proteins is generally achieved by COPII-coated vesicles, which comprise four coat proteins, namely Sec23, Sec24, Sec13, and Sec31^14^. Assembly of the COPII coat on the ER membrane is initiated by the activation of the small GTPase Sar1 via its guanine nucleotide exchange factor Sec12. Activated Sar1 recruits the COPII inner layer Sec23/Sec24 complex and subsequently the outer layer Sec13/Sec31 complex to induce the packaging of client cargo and deform the ER membrane to produce transport vesicles^15–17^. In this process, various ER transmembrane proteins function as specific cargo receptors to facilitate the ER export of specific cargoes by concentrating them at the ER exit sites (ERES), where COPII vesicles are actively formed^16–23^.

Particularly, Surf4 is a mammalian homolog of Erv29p, which was originally identified as a multi-spanning transmembrane cargo receptor for a yeast sex pheromone precursor, pro-α-factor, and a vacuolar protease, carboxypeptidase Y (CPY), in *Saccharomyces cerevisiae*^24^. However, Surf4 had been thought to be dispensable for general protein secretion in mammalian cells as the total amount of secreted proteins was neither affected by its single knockdown nor its simultaneous knockdown with another cargo receptor ERGIC-53 in HeLa cells^25^. Recent studies have revealed that Surf4 facilitates the ER export of specific cargoes, including very-low-density lipoprotein (VLDL), proprotein convertase subtilisin/kexin type 9 (PCSK9), growth hormone (GH), dentin sialophosphoprotein (DSPP), amelogenin X-linked (AMELX), and erythropoietin^26–31^. In addition, it has been reported that Surf4 is detected in COPII vesicles in an in vitro budding assay, suggesting that Surf4 itself enters the COPII vesicles like yeast Erv29p^32^.

Here, we show that Surf4 plays a critical role in ER export of proinsulin. Surf4 was highly expressed in the pancreas and upregulated by high-glucose stimulation in rat pancreatic β cell lines. Surf4 mainly localizes to the ER at a steady state and the protein level of Surf4 at the ERES increased under high-glucose condition. Loss of Surf4 in rat pancreatic β cell lines caused the accumulation of proinsulin in the ER, resulting in severe inhibition of glucose-stimulated insulin secretion. Surf4 physically interacted with proinsulin and Sec12, and loss of Surf4 affected the organization of the ERES. These findings suggest that Surf4 functions as a cargo receptor that recruits proinsulin to the ERES for efficient ER export of proinsulin in pancreatic β cells.

## Results

### Surf4 expression is upregulated in glucose-stimulated pancreatic β cells

**Fig. 1:**
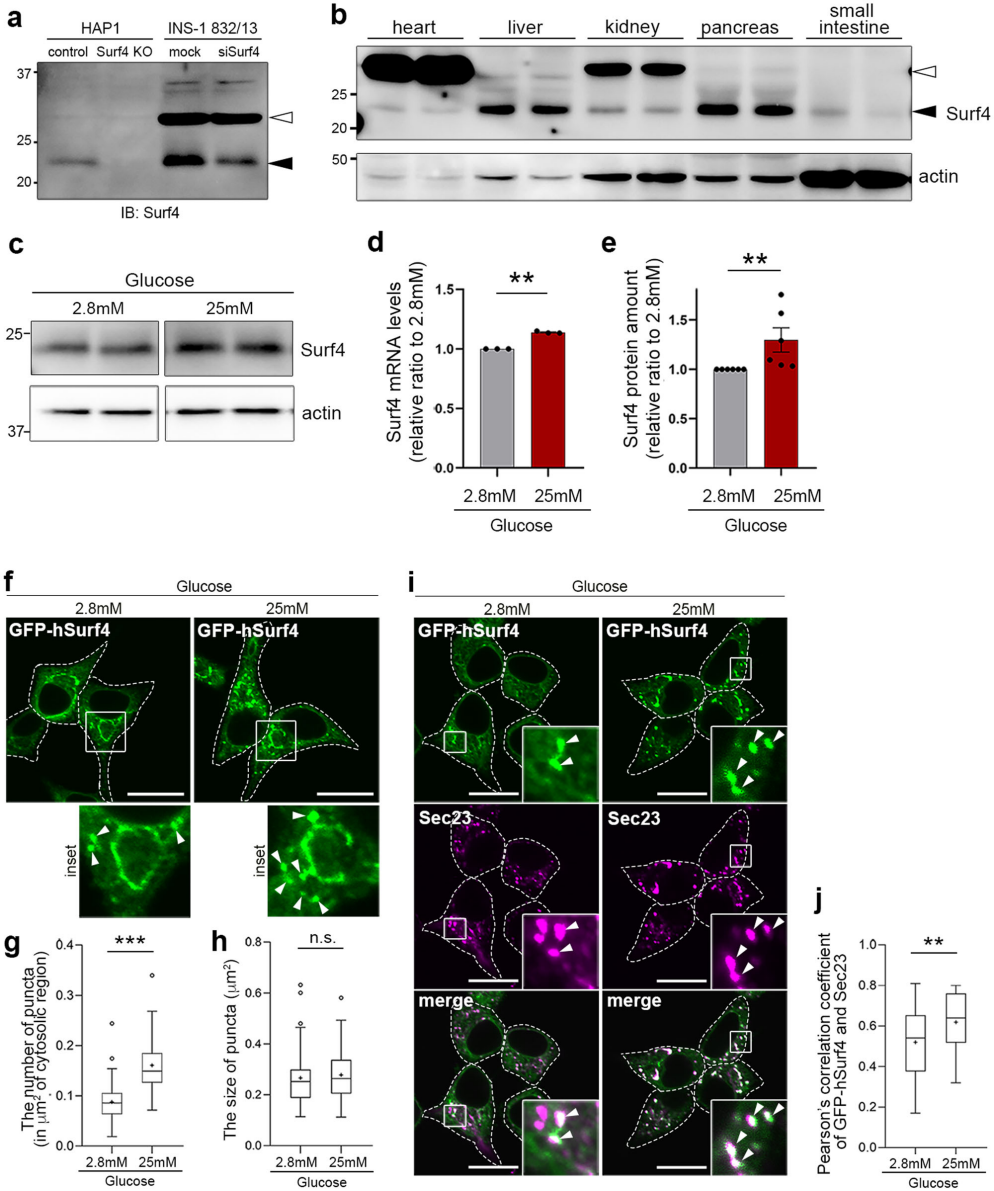
Surf4 expression is upregulated in glucose-stimulated pancreatic β cells. **a** Whole-cell lysates of HAPl and INS-1 832/13 cells were immunoblotted with an antibody against Surf4. Surf4 protein in control cells was detected as an approximately 23 kDa molecular weight band (black arrowhead), which disappeared in Surf4-deficient cells and weakened in Surf4 siRNA-treated cells. A white arrowhead indicates a non­specific detection. **b** Tissue-expression pattern of Surf4 in mice. Mice tissue lysates (10 µg of protein per lane) were subjected to immunoblotting analysis using an anti-Surf4 and anti-β-actin antibodies. **c** Whole-cell lysates of INS-1 832/13 cells incubated with Krebs–Ringer buffer (KRB) containing low (2.8 mM) or high (25 mM) glucose for 2 h were subjected to immunoblotting analysis using anti-Surf4 and anti-β-actin antibodies. **d**, **e** Quantification of Surf4 mRNA (**d**; *n* = 3) and protein levels (**e**; *n* = 6) of INS-1 832/13 cells following low- or high-glucose simulation. Data are presented as the mean ± standard error of the mean and analyzed by two-tailed Welch’s *t*-test (**d**) and two-tailed Mann–Whitney *U* test (**e**), **p < 0.05. **f** Subcellular localization of GFP-human Surf4 (hSurf4) in INS-1 832/13 cells following low- or high-glucose simulation. Arrowheads indicate GFP-hSurf4 accumulation. Dotted lines indicate the outlines of the cells. Scale bars: 10 µm. **g**, **h** The number and size of GFP-hSurf4-positive punctate structures in (**f**) were quantified (*n* = 55, low-glucose-treated cells; *n* = 57, high-glucose treated cells). Data are presented as the box and whisker plots and analyzed by two-tailed Mann–Whitney *U* test (**g**) and two-tailed unpaired Student’s *t*-test (**h**), ***p < 0.01; n. s. not significant. **i** Subcellular localization of GFP-hSurf4 and Sec23 in INS-1 832/13 cells following low- or high-glucose simulation. White arrowheads indicate GFP-hSurf4-positive punctate structures that overlap with Sec23. Dotted lines indicate the outlines of the cells. Scale bars: 10 µm. **j** Quantification of the colocalization of GFP-hSurf4 and Sec23 puncta as calculated using Pearson’s correlation coefficient (*n* = 40, low-glucose-treated cells; *n* = 34, high-glucose-treated cells). Data are presented as the box and whisker plots and analyzed by two-tailed unpaired Student’s *t*-test, **p < 0.05.

We first examined the expression of Surf4 in human HAP1 cells and rat insulinoma INS-1 832/13 cells. Although the estimated molecular weight of Surf4 was approximately 30 kD, we found that a protein at an approximately 23 kD was completely abolished in *Surf4*-deleted HAP1 cells and reduced in amount in Surf4(siRNA)-treated INS-1 832/13 cells (Fig. 1a). We also confirmed that the amount of the protein at 23 kD was reduced in INS-1 832/13 cells, which was treated with a different siRNA against rat Surf4 (oligo#2), suggesting that this protein represents endogenous Surf4 (Supplementary Fig. 1a, b). In tissues of C57BL/6N mice, Surf4 proteins were expressed in the pancreas and liver but weakly expressed in the heart, kidney, and small intestine (Fig. 1b).

As Surf4, a mammalian homolog of yeast Erv29p, was highly expressed in the pancreas, we speculated that it is involved in the mechanism underlying hormone secretion by pancreatic endocrine cells. Thus, we examined whether *Surf4* expression was induced by high-glucose stimulation. Interestingly, high-glucose stimulation significantly increased the levels of both Surf4 mRNA and protein by approximately 1.1- and 1.3-folds compared with those in cells treated with low glucose, respectively (Fig. 1c–e).

In addition, we examined the subcellular localization of Surf4 following glucose stimulation. In low-glucose conditions, a human Surf4 with an N-terminal GFP tag (GFP-hSurf4) mostly localized to typical ER reticular structures, which were immunostained with an anti-PDI antibody (Fig. 1f and Supplementary Fig. 1c). GFP-hSurf4 was also detected with some punctate structures, which colocalized with Sec23, a marker of ERES, by immunofluorescent microscopy, suggesting that Surf4 partly localizes to the ERES under low-glucose condition (Fig. 1i). A part of GFP-hSurf4-puncta was observed in a crescent shape region, adjacent to ERGIC-53-positive ERGIC and GM130-positive cis-Golgi but not with TGN38-labeled TGN (Supplementary Fig. 1d–f). Interestingly, the number of GFP-hSurf4-positive punctate structures was significantly increased by high-glucose stimulation compared with low-glucose conditions; however, their sizes remained unchanged (Fig. 1f–h). Most GFP-hSurf4 punctate structures induced by high-glucose conditions colocalized with Sec23, suggesting that GFP-hSurf4 localizes to the ERES (Fig. 1i, j). These results indicate that in high-glucose conditions, Surf4 is upregulated and predominantly localizes to the ERES in pancreatic β cells.

### Surf4 is required for insulin secretion

**Fig. 2:**
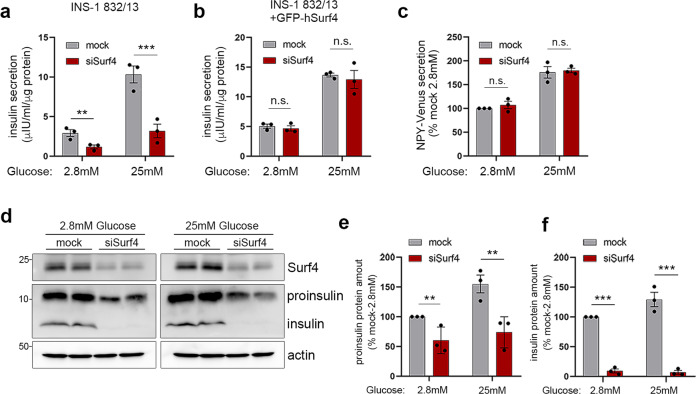
Surf4 is required for insulin secretion. **a**, **b** INS-1 832/13 cells (**a**) or INS-1 832/13 cells stably expressing GFP-hSurf4 (**b**) were transfected with siRNA against non-target control (mock) or rat Surf4 (siSurf4) and incubated with KRB containing low (2.8 mM) or high (25 mM) glucose for 2 h. The amount of secreted insulin was measured (*n* = 3). **c** INS-1 832/13 cells stably expressing a neuropeptide Y (NPY)-Venus were treated with mock- or Surf4-siRNA and incubated at low- or high-glucose conditions. The amount of secreted NPY-Venus was measured (*n* = 3). **d** Whole-cell lysates of INS-1 832/13 incubated at low- or high-glucose conditions were subjected to immunoblotting analysis using an anti-insulin antibody that recognizes both proinsulin and insulin and anti-Surf4, anti-β-actin antibodies. **e**, **f** Proinsulin and insulin levels in (**d**) were quantified by densitometric scanning of band intensities, and their relative amounts were calculated (*n* = 3). Data are presented as the mean ± standard error of the mean and analyzed by two-tailed unpaired Student’s *t*-tests (**a**–**c**, **e**, **f**), **p < 0.05; ***p < 0.01; n.s. not significant.

As Surf4 expression was upregulated in high-glucose-stimulated pancreatic β cells, we hypothesized that Surf4 is involved in glucose-stimulated insulin secretion. We found that the loss of Surf4 markedly inhibited insulin secretion, especially in cells stimulated with high glucose (Fig. 2a). This reduction was restored by expressing GFP-tagged human Surf4, a sequence that is resistant to siRNA against rat Surf4, thus indicating that the impairment of insulin secretion was not due to off-target effects (Fig. 2b and Supplementary Fig. 2a). To further evaluate the efficiency of siRNA against rat Surf4, we knocked down rat Surf4 gene in INS-1 832/13 cells using a second siRNA oligo targeting rat Surf4 (oligo#2) and found that the mRNA levels of Surf4 was significantly reduced (Supplementary Fig. 2b). In addition, the secreted insulin amounts were significantly decreased when Surf4 was silenced using siSurf4 (oligo#2), indicating that the impairment of insulin secretion resulted from the loss of Surf4 (Supplementary Fig. 2c). Moreover, we found that the secretion of neuropeptide Y tagged with Venus at its C-terminus (NPY-Venus), which is transported into the secretory granules and exocytosed along with insulin^33,34^, was not significantly affected in Surf4(siRNA)-treated cells (Fig. 2c). These results indicate that insulin secretion was specifically impaired by the loss of Surf4. We also measured remaining insulin levels and observed decreased subcellular insulin and proinsulin levels in Surf4 siRNA-treated cells under low- and high-glucose conditions (Fig. 2d–f). However, mRNA levels of *ins-1* were comparable between mock and Surf4 siRNA-treated cells (Supplementary Fig. 2d, e).

### Surf4 is required for the ER export of proinsulin

**Fig. 3:**
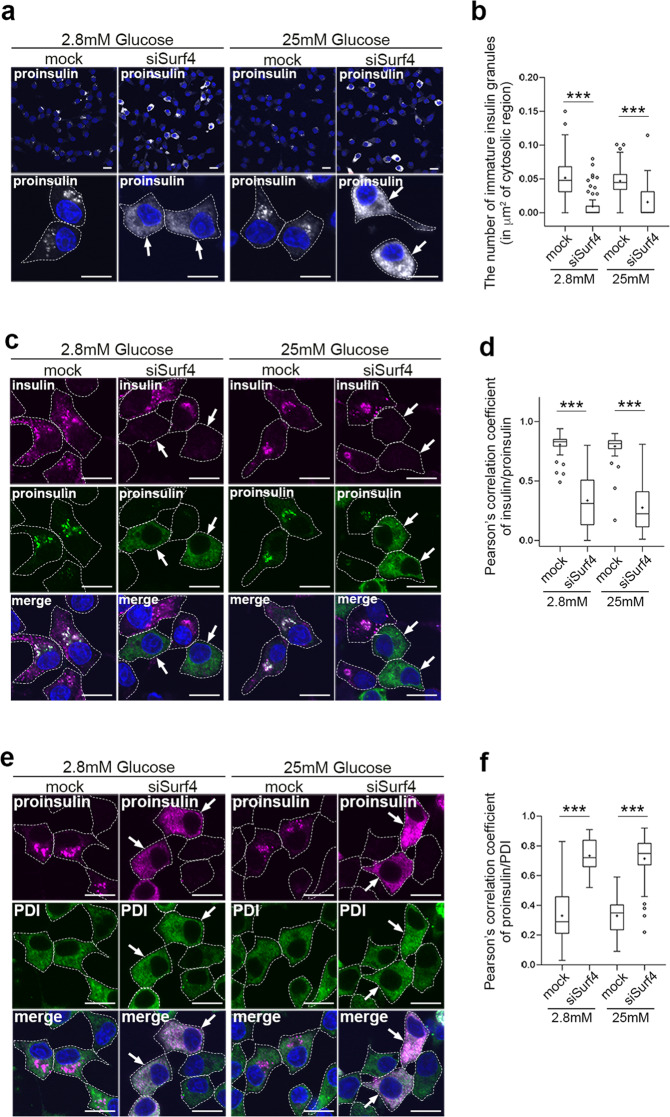
Surf4 is required for the ER export of proinsulin. **a** INS-1 832/13 cells were fixed and stained with an anti-proinsulin antibody, which specifically recognize proinsulin. Arrows indicate cells showing proinsulin accumulated in reticular structures. Dotted lines indicate cell outlines. Scale bars: 10 µm. **b** The number of immature insulin granules containing proinsulin in the cytoplasm was quantified (*n* = 60, mock-transfected cells at low-glucose conditions; *n* = 70, siSurf4-transfected cells at low-glucose conditions; *n* = 56, mock-transfected cells at high-glucose conditions; *n* = 59, siSurf4-transfected cells at high-glucose conditions). **c** INS-1 832/13 cells were fixed and stained with anti-insulin and anti-proinsulin antibodies. Arrows indicate cells that show the decreased number of insulin-positive granules and the accumulation of proinsulin in reticular structures. Dotted lines indicate cell outlines. Scale bars: 10 µm. **d** Quantification of colocalization of insulin and proinsulin were calculated by Pearson’s correlation coefficient (*n* = 58, mock-transfected cells at low-glucose conditions; *n* = 55, siSurf4-transfected cells at low-glucose conditions; *n* = 62, mock-transfected cells at high-glucose conditions; *n* = 56, siSurf4-transfected cells at high-glucose conditions). **e** INS-1 832/13 cells were fixed and stained with anti-proinsulin and anti-PDI antibodies. Arrows indicate cells that show the colocalization of proinsulin with an ER marker protein, PDI. Dotted lines indicate cell outlines. Scale bars: 10 µm. **f** Quantification of colocalization of proinsulin and PDI were calculated by Pearson’s correlation coefficient (*n* = 51, mock-transfected cells at low-glucose conditions; *n* = 42, siSurf4-transfected cells at low-glucose conditions; *n* = 45, mock-transfected cells at high-glucose conditions; *n* = 41, siSurf4-transfected cells at high-glucose conditions). Data are presented as the box and whisker plots and analyzed by two-tailed Mann–Whitney *U* tests, ***p < 0.01.

**Fig. 4:**
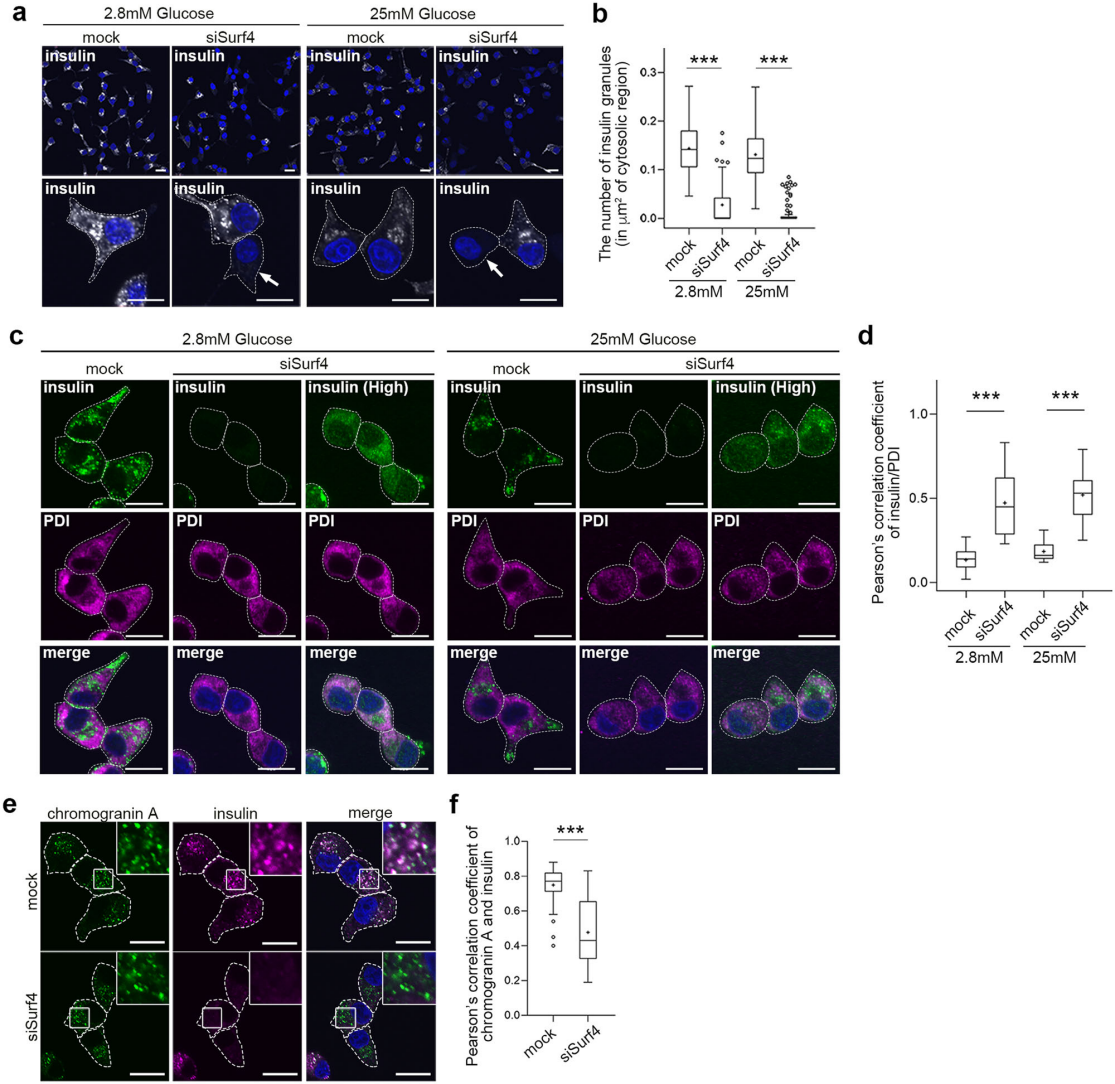
Loss of Surf4 decreased the number of insulin-positive granules. **a** INS-1 832/13 cells transfected with mock or Surf4 siRNA(siSurf4) were incubated in KRB containing low (2.8 mM) or high (25 mM) glucose for 2 h. Cells were fixed and stained with an anti-insulin antibody which recognizes mature insulin and weakly reacts with proinsulin. Arrows indicate Surf4-siRNA transfected cells with reduced insulin-positive granules. Dotted lines indicate cell outlines. Scale bars: 10 µm. **b** The number of insulin granules in the cytoplasm was quantified (*n* = 67, mock-transfected cells at low-glucose conditions; *n* = 73, siSurf4-transfected cells at low-glucose; *n* = 62, mock-transfected cells at high-glucose conditions; *n* = 69, siSurf4-transfected cells at high-glucose conditions). **c** INS-1 832/13 cells were transfected with the mock- or Surf4-siRNA and incubated in KRB containing low (2.8 mM) and high (25 mM) glucose concentrations for 2 h. Cells were fixed and stained with anti-insulin and anti-PDI antibodies. The fluorescent signals of insulin in siSurf4 cells were detected with the same laser power to observe mock-treated cells (middle panels) or an approximately 1.4-fold higher laser power (right panels). Dotted lines indicate cell outlines. Scale bars: 10 µm. **d** Quantification of the colocalization of insulin (insulin (High) in **e**) and PDI as calculated by Pearson’s correlation coefficient (*n* = 17, mock­transfected cells at low-glucose conditions; *n* = 24, siSurf4-transfected cells at low-glucose conditions; *n* = 13, mock-transfected cells at high-glucose conditions; *n* = 22, siSurf4-transfected cells at high-glucose conditions). **e** INS-1 832/13 cells that were transfected with mock or siSurf4 were fixed and immunostained with anti-chromogranin A and anti-insulin antibodies. Dotted lines indicate cell outlines. Scale bars: 10 µm. **f** Quantification of the colocalization of chromogranin A and insulin-positive puncta as calculated using Pearson’s correlation coefficient (*n* = 46, mock cells; *n* = 41, siSurf4 cells). Data are presented as the box and whisker plots and analyzed by two-tailed Mann–Whitney *U* tests, ***p < 0.01.

We further examined the subcellular localization of insulin by immunostaining. To test this, we used an anti-proinsulin antibody that specifically recognizes proinsulin but not mature insulin and an anti-insulin antibody that recognizes mature insulin and weakly reacts with proinsulin^35^. Anti-insulin antibody stained two populations of the subcellular granular structures: one localized to the center region of cells (Supplementary Fig. 3a, arrows) and another localized to the cell periphery (Supplementary Fig. 3a, arrowheads). Notably, the former granular structures were also stained with anti-proinsulin antibody (Supplementary Fig. 3a, arrowheads), indicating that these granules were immature insulin granules that contain proinsulin. The latter granular structures observed in the cell periphery represented mature insulin granules containing mature insulin after processing. We first examined the subcellular localization of proinsulin in Surf4-depleted cells by immunostaining using an anti-proinsulin antibody (Fig. 3). The signal of proinsulin was mainly observed in immature insulin granules, which were located close to the Golgi apparatus, in mock-treated cells at a steady state (Supplementary Fig. 4a, b). By contrast, it was largely redistributed to the ER-like reticular structure in Surf4 (siRNA)-treated cells (Fig. 3a, b, and Supplementary Fig. 4b). Notably, Surf4 (siRNA)-treated cells showed reduced number of insulin-positive granules and reticular localization of proinsulin (Fig. 3c, d). In such cells, proinsulin was largely colocalized with PDI, but not with ERGIC-53, suggesting that proinsulin was retained in the ER luminal region (Fig. 3e, f, and Supplementary Fig. 4b, c). These results indicate that Surf4 is required for the export of proinsulin from the ER. We also found that the number of insulin- and proinsulin-positive secretory granules were significantly reduced in Surf4(siRNA)-treated cells under both low- and high-glucose conditions (Fig. 4a, b). As fluorescent signals by immunostaining using anti-insulin antibody, which recognizes mature insulin and weakly reacts with proinsulin, were attenuated in Surf4-depleted cells, we further observed the insulin signals with an approximately 1.4-fold higher laser power (Fig. 4c, d). Under this condition, the insulin signals were detected on reticular structures, which were colocalized with PDI, suggesting that the loss of Surf4 caused accumulation of proinsulin in the ER (Fig. 4c, d). To examine whether secretory granules were disappeared in Surf4 (siRNA)-treated cells, we observed the localization of chromogranin A, which is transported to the plasma membrane together with insulin via insulin granules (Fig. 4e). Insulin signals were mainly detected in granular structures that largely overlapped with chromogranin A-positive secretory granules in mock cells^36–39^. By contrast, although chromogranin A-positive secretory granules were still observed in Surf4 (siRNA)-treated cells, the insulin signals mostly disappeared from these granules (Fig. 4e, f). We confirmed that the expression of GFP-hSurf4 restored the disappearance of insulin-positive granules due to Surf4 depletion (Supplementary Fig. 3b, c). These results suggest that although the organization of chromogranin A-positive secretory granules occurred to some extent, proinsulin was not appropriately targeted to the secretory granules.

### Surf4 physically interacts with proinsulin

We then determined whether Surf4 physically interacts with proinsulin in the ER to confirm its function as a sorting receptor for proinsulin. To test this, we used human preproinsulin tagged with mRFP at its C-terminus (hInsulin-mRFP)^40–42^. We found that hInsulin-mRFP localized to many granular structures that overlapped with insulin, proinsulin or chromogranin A at a steady state, indicating that hInsulin-mRFP is properly synthesized and transported into secretory granules (Supplementary Fig. 5a–c).

**Fig. 5:**
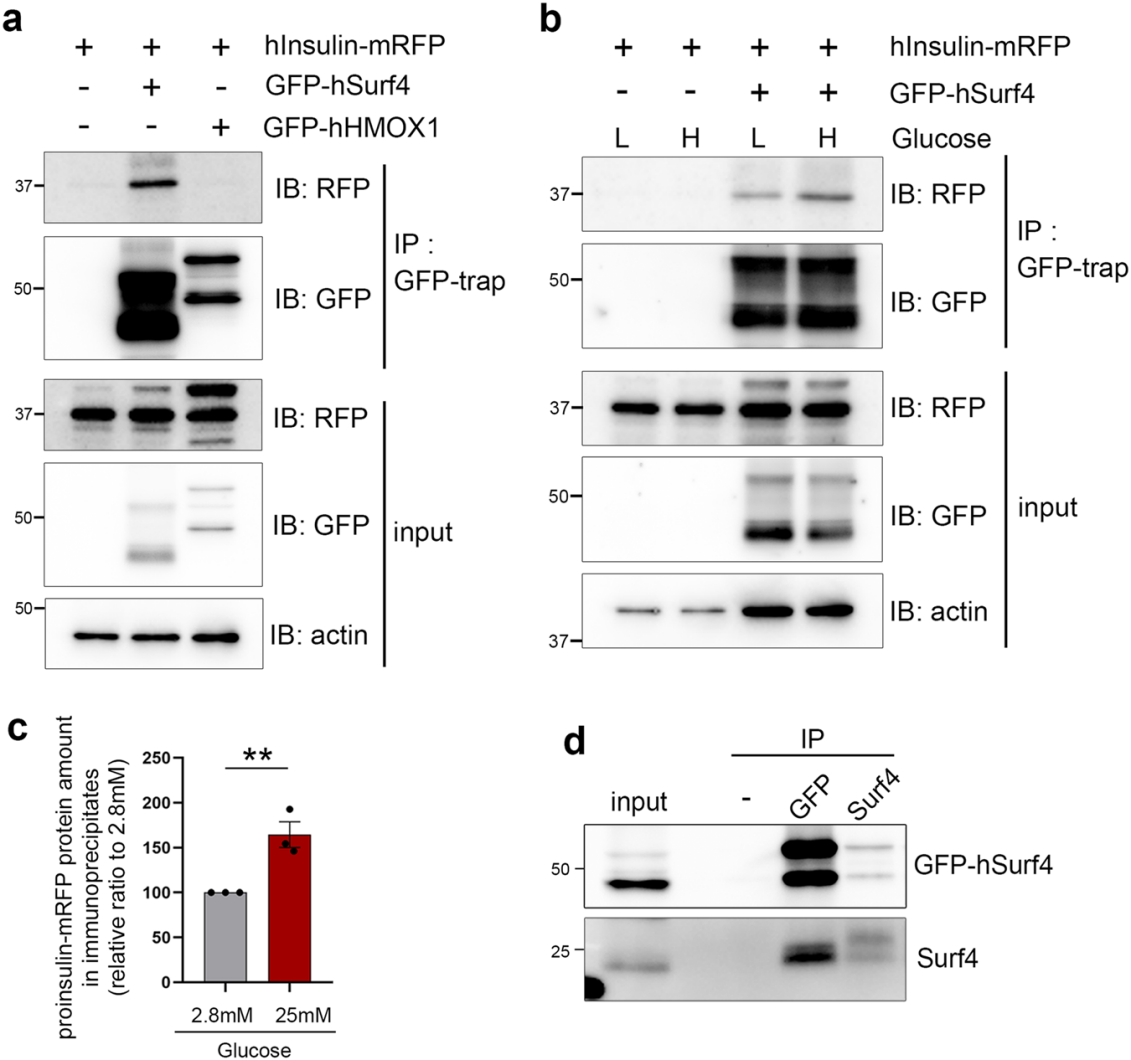
Surf4 physically interacts with proinsulin. **a** INS-1 832/13 cells expressing human Insulin (hInsulin)-mRFP and GFP-human Surf4 (hSurf4) or GFP-human HMOXl (hHMOX1) were immunoprecipitated with a GFP-trap. Immunoprecipitates (IP) and 0.2% of the total lysate (input) were immunoblotted with anti-RFP, anti-GFP, and anti-β-actin antibodies. An approximately 37 kDa protein, which was predicted to be a proinsulin-mRFP, was co-immunoprecipitated with GFP-hSurf4 but not with GFP-hHMOX1. **b** INS-1 832/13 cells expressing hInsulin-mRFP and GFP-hSurf4 were incubated in KRB containing low- (L: 2.8 mM) or high- (H: 25 mM) glucose for 15 min. Cells were then immunoprecipitated with the GFP-trap. Immunoprecipitates (IP) and 0.2% of the total lysate (input) were immunoblotted with anti-RFP, anti-GFP, and anti-β-actin antibodies. **c** Quantification of hInsulin-mRFP protein amount contained in immunoprecipitates following low- or high-glucose stimulation (**b**; *n* = 3). Data are presented as the mean ± standard error of the mean and analyzed by two-tailed *t*-test, **p < 0.05. **d** INS-1 832/13 cells expressing GFP-hSurf4 were immunoprecipitated with anti-GFP and anti-Surf4 antibodies. IP and 1% of the total lysate (input) were immunoblotted with anti-Surf4 and anti-GFP antibodies.

Co-immunoprecipitation experimental results showed that proinsulin-mRFP was immunoprecipitated with GFP-hSurf4 but not with GFP-tagged human heme oxidase-1 (GFP-hHMOX1), an ER membrane protein (Fig. 5a and Supplementary Fig. 5d). Interestingly, a high-glucose treatment increased the amount of proinsulin-mRFP, which was co-immunoprecipitated with GFP-hSurf4 (Fig. 5b, c). GFP-hSurf4 was co-immunoprecipitated with endogenous Surf4, suggesting that Surf4 functions as an oligomer in vivo (Fig. 5d). These results suggest that Surf4 physically interacts with proinsulin at the ERES and mediates its packaging into transport vesicles.

### Surf4 affects the size and number of the ER exit sites

**Fig. 6:**
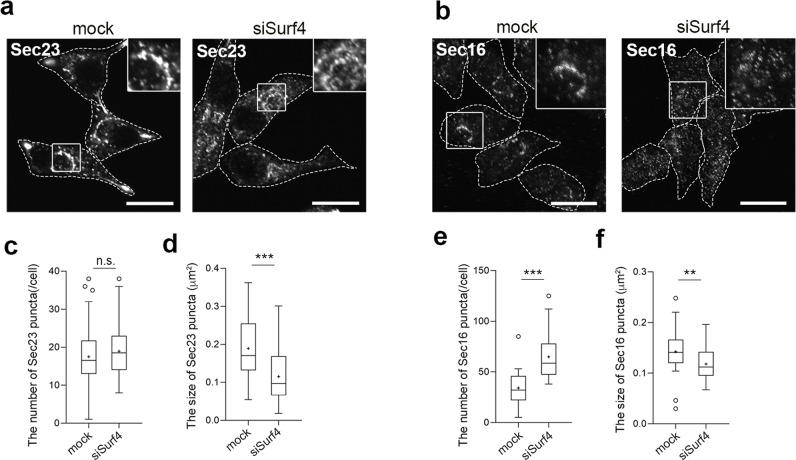
Loss of Surf4 affects the number and size of the ERES in pancreatic β cells. **a** INS-1 832/13 cells transfected with the mock or siSurf4 siRNA were fixed and immunostained with an anti-Sec23 antibody. Dotted lines indicate cell outlines. Scale bars: 10 µm. **b** INS-1 832/13 cells transfected with the mock or siSurf4siRNA were fixed and immunostained with an anti-Secl6 antibody. Dotted lines indicate cell outlines. Scale bars: 10 µm. **c**, **d** Quantification of the number and size of Sec23-positive punctate structures (*n* = 52, mock cells; *n* = 50, siSurf4 cells). Data are presented as the box and whisker plots and analyzed by two-tailed Mann–Whitney *U* test, ***p < 0.01; n.s. not significant. **e**, **f** Quantification of the number and size of Sec16-positive punctate structures (*n* = 31, mock cells; *n* = 32, siSurf4 cells). Data are presented as the box and whisker plots and analyzed by two-tailed Mann–Whitney *U* test, **p < 0.05, ***p < 0.01.

We further examined the effect of Surf4 depletion on the organization of the ERES, wherein proinsulin is packaged into COPII-coated vesicles in pancreatic β-cell lines. Packaging of cargo proteins into COPII vesicles is initiated by the activation of the small GTPase Sar1 via its specific GEF, Sec12. Activated Sar1 then recruits Sec23/Sec24 and Sec13/Sec31 to form COPII vesicles at the ERES^15–17^. Sec23, the inner layer of the COPII coat^43^, was mainly detected on many punctate structures, some of which were distributed in a crescent-shaped region that largely overlapped with GFP-hSurf4 in mock-treated cells (Figs. 6a and 1i). In contrast, the loss of Surf4 caused the redistribution of Sec23 to smaller punctate structures (Fig. 6a). We also examined whether the loss of Surf4 affected the localization of Sec16, a peripheral membrane protein that functions as a scaffold for COPII vesicle assembly and a platform for regulating ERES formation^44^. Sec16 was also distributed in a crescent-shaped region in mock-treated cells; however, in Surf4(siRNA)-treated cells, its localization was redistributed to smaller punctate structures, along with that of Sec23 (Fig. 6b). The numbers of Sec16-positive punctate structures were increased and the size of Sec23- and Sec16-positive punctate structures were significantly decreased in Surf4-depleted cells (Fig. 6c–f), suggesting that the Surf4 affects the property of the ERES.

**Fig. 7:**
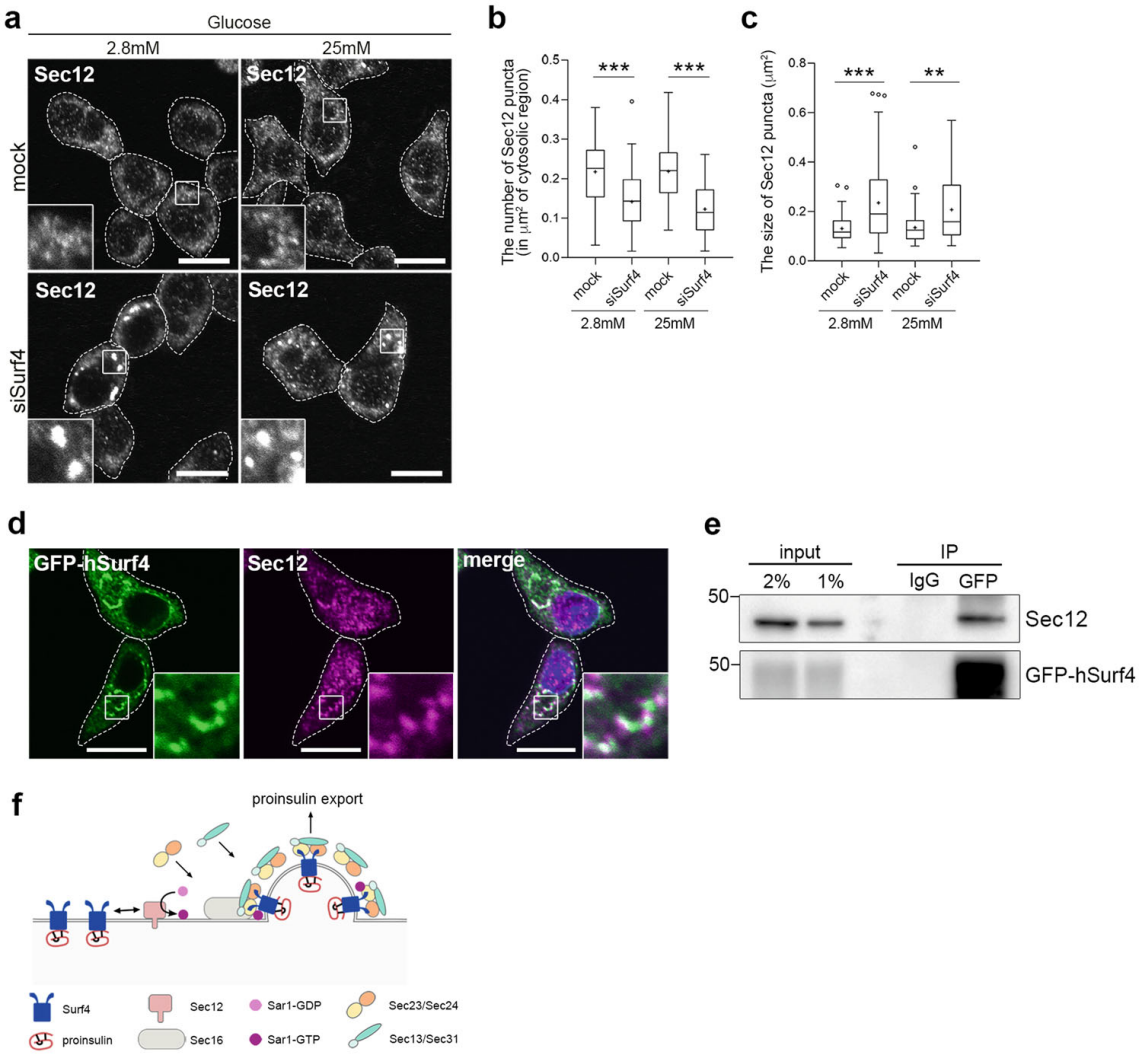
Surf4 physically interacts with Sec12 and affects its localization. **a** INS-1 832/13 cells were transfected with the control (mock) or Surf4 siRNA(siSurf4) and incubated in KRB containing low (2.8 mM) or high (25 mM) glucose for 2 h. Cells were fixed and stained with an anti-Sec12 antibody. Dotted lines indicate cell outlines. Scale bars: 10 µm. **b**, **c** The number and size of Sec12-positive punctate structures in the cytoplasm were measured (*n* = 86, mock-transfected cells at low-glucose conditions; *n* = 83, siSurf4-transfected cells at low-glucose conditions; *n* = 73, mock-transfected cells at high-glucose conditions; *n* = 71, siSurf4-transfected cells at high-glucose conditions). Data are presented as the box and whisker plots, and analyzed by two-tailed Mann–Whitney *U* tests, **p < 0.05; ***p < 0.01. **d** INS-1 832/13 cells expressing GFP-hSurf4 were immunostained with an anti-Sec12 antibody. GFP-Surf4 partially colocalizes with Sec12. Dotted lines indicate cell outlines. Scale bars: 10 µm. **e** GFP-hSurf4 physically interacts with Sec12. INS-1 832/13 cells expressing GFP-hSurf4 were immunoprecipitated with an anti-GFP antibody. Total lysates (input, 2 and 1%) and Immunoprecipitates (IP) were immunoblotted with anti-Secl2 and anti-GFP antibodies. **f** Schematic of the proposed function of Surf4 in the ER export of proinsulin.

Next, we investigated whether the loss of Surf4 affects the subcellular localization of Sec12. According to immunostaining results, Sec12 localized to small punctate structures in mock-treated INS-1 832/13 cells at a steady state (Fig. 7a). Notably, the loss of Surf4 reduced the number of Sec12-positive punctate structures and the redistribution of Sec12 to enlarged punctate structures under both low- and high-glucose conditions (Fig. 7a–c); however, its protein level remain unchanged (Supplementary Fig. 6a, b). We also confirmed that Sec12-positive large punctate structures were formed in *Surf4*-deleted human HAP1 cells but not in the control cells (Supplementary Fig. 6c). These results suggest that Surf4 is involved in the proper localization of Sec12 in INS-1 832/13 cells. Indeed, a part of GFP-hSurf4 were colocalized with Sec12 particularly on crescent-shaped region of the ER in INS-1 832/13 cells at a steady state (Fig. 7d). We also examined a physically interaction between Surf4 and Sec12. We found that GFP-hSurf4 was co-immunoprecipitated with endogenous Sec12, suggesting that Surf4 directly or indirectly interacts with Sec12 (Fig. 7e). Collectively, we demonstrated that Surf4 functions in the sorting of proinsulin into the COPII vesicles and also modulates the proper distribution of Sec12, Sec23, and Sec16 (Fig. 7f).

## Discussion

Insulin regulates the overall blood glucose concentration, and defects in insulin biogenesis and secretion are associated with the onset of diabetes and related diseases. Although the mechanisms underlying insulin exocytosis have been studied well, our knowledge of the molecular mechanisms underlying the intracellular trafficking of proinsulin remains limited, particularly with regard to how nascent proinsulin are concentrated in the vesicles and efficiently exported from the ER. In this study, we demonstrated that Surf4 is required for the ER export of proinsulin in pancreatic β cells. Upon its depletion, the export of proinsulin from the ER was strongly inhibited as evidenced by significantly decreased glucose-stimulated insulin secretion. We also found that Surf4 predominantly localizes to the ERES under high-glucose conditions and physically interacts with proinsulin. These findings suggest that Surf4 functions as a sorting receptor for the efficient export of proinsulin from the ER. Surf4 expression is upregulated by high-glucose stimulation, leading to its redistribution to the ERES. Moreover, we found that Surf4 forms homo-oligomers in vivo and physically interacts with proinsulin and Sec12, a specific GEF of Sar1 that initiates the assembly of COPII coat proteins at the ER membrane. In INS-1 832/13 cells, a part of Surf4 colocalized with Sec12 and Sec23 on the crescent-shaped regions of the ER, which may be active ERESs for proinsulin. Upon *Surf4* knockdown, Sec12 was redistributed to large round structures, while Sec23 and Sec16 became localized to smaller punctate structures. From these observations, a transient interaction between Surf4 and Sec12 may promote the accommodation of proinsulin into COPII vesicles and form enlarged ERES zones, which efficiently export proinsulin from the ER in INS-1 832/13 cells.

As the loss of Surf4 affected Sec12 localization and caused the redistribution of Sec23 and Sec16, we assumed that Surf4 would be required for the general processes of ER export of secretory proteins. However, although Surf4 is critical for the ER exits of proinsulin, the secretion of Venus-tagged NPY and the trafficking of chromogranin A occurred to some extent, which suggests that Surf4 is not necessarily essential for the general processes operating in the early secretory pathway. These results raise the possibility that there are ERESs specialized for the trafficking of proinsulin in insulin-producing β cells. Previously, it was reported that mammalian ER exit sites are observed as structures with approximately 400 nm in size that contain several COPII-coated structures on average^45,46^. Interestingly, the punctate structures labeled by Sec23 and Sec16 in INS-1 832/13 cells were relatively larger than that in cells investigated so far^14,16,18–20,45^, suggesting that these large structures indicated the atypical ERESs constituted by a number of ERES component proteins that specialized for the proinsulin ER export. Therefore, because Surf4 depletion in pancreatic β cells caused the disruption of these large ERESs, the ER export of massive amount of proinsulin was specifically inhibited.

Co-immunoprecipitation experimental results showed that Surf4 physically interacts with proinsulin, however, it is not certain whether this interaction was direct or indirect. Because Surf4 can form complexes with various transmembrane proteins, including ERGIC-53, p24δ1 (also known as p23 or TMED10), and p24β1 (also known as p24 or TMED2)^25^, it is possible that Surf4 indirectly binds to proinsulin and requires other transmembrane proteins that could link Surf4 and proinsulin for the enrichment and loading of proinsulin into COPII-coated vesicles. p24δ1, a member of the p24 family proteins, has been reported to be required for insulin biogenesis and secretion in β cells as its gene knockdown reduced cellular insulin content and impaired glucose-stimulated insulin secretion^47^. Therefore, p24δ1 and Surf4 may participate in the formation of large ERES domains on the ER membrane to export massive amounts of nascent proinsulin from the ER under high-glucose conditions.

These results demonstrated that Surf4 regulates the ER export of small peptide prohormones, in addition to macromolecules such as lipid-binding lipoproteins VLDLs^26,31^. Increasing evidence indicates that Surf4 is required for the ER export of various cargoes, suggesting that Surf4 can recognize diverse soluble secretory proteins depending on cell function. Indeed, Surf4 is essential for early development in animals. Its homozygous knockout caused embryonic lethality in both *Caenorhabditis elegans* and mice^26,48^, suggesting that Surf4 plays a broader role in protein secretion.

Interestingly, a genome-wide database of the International Mouse Phenotyping Consortium (IMPC) showed that heterozygous male mice with the *Surf4*^*em1(IMPC)J*^ allele, which causes the deletion of exon 2, exhibit impaired glucose tolerance, a symptom of diabetes mellitus in patients with maturity-onset diabetes of the young and type 1 diabetes^49^. This implies that the loss-of-function of *Surf4* in β cells might increase the risk for the onset and progression of diabetes. We revealed that Surf4 function as a limiting factor for insulin secretion, implying that drugs that upregulate the Surf4 expression, may promote the insulin secretion by enhancing the ER export of proinsulin, and improve high blood glucose levels. How Surf4 regulates the organization of complexes comprising proinsulin, ERES components, and other potential cofactors under high-glucose conditions should be investigated to provide insights into the mechanisms of ER–Golgi trafficking during insulin secretion and to identify new targets for the treatment of diabetes accompanied by decreased glucose-stimulated insulin secretion.

## Materials and methods

### Mice

All animal experiments were approved by the Animal Care and Experimentation Committee of Gunma University and were performed in accordance to its guidelines. The approval number for this study was 16-004. The C57BL/6 N mice were bred with standard diet and the 8-week old male mice were anesthetized with tribromoethanol (Avertin; Sigma-Aldrich, Tokyo, Japan) by intraperitoneal injection and perfused intracardially with phosphate-buffered saline (PBS). The isolated tissues were frozen in liquid nitrogen and stored at −80 °C.

### Cell lines

Rat insulinoma INS-1 832/13 cells^50^ were provided by Dr. C. Newgard (Duke University, Durham, NC, USA) and cultured in RPMI 1640 medium containing 10% fetal bovine serum (FBS) supplemented with 2 mM L-glutamine, 10 mM HEPES, 1 mM sodium pyruvate, and 0.05 M 2-mercapthoethanol. INS-1 832/13 cells stably expressing *GFP-hSurf4* or *NPY-Venus* were generated via retroviral transfection. Production of retrovirus and retroviral infection was performed as described previously^51,52^. INS-1 832/13 cells were infected with the GFP-human Surf4/pMXs-IP or the NPY-Venus/pMRX-IRES-puro-DEST virus and selected with 1 µg/mL puromycin. Human haploid cell lines (HAP1 cells)^53,54^ were obtained from Horizon Discovery (Cambridge, UK). *Surf4*-deficient HAP1 cell line, which contains a 13-bp deletion and a frameshift mutation in exon 4 (Surf4 KO; HAP1_SURF4_02492-01), and the control HAP1 strain (# C665) were used. HAP1 cells were cultured in Iscove’s modified Dulbecco’s medium supplemented with 10% FBS. All cells were incubated at 37 °C in a humidified atmosphere containing 95% air and 5% CO_2_.

### Plasmids

To construct pMXs-IP-GFP-human Surf4 and pcDNA3.1-GFP-human HMOX1, cDNA fragments of human Surf4 and HMOX1 were PCR-amplified from reverse-transcribed total RNA of HepG2 cells and subcloned into the entry vector pDONR221 using Gateway Recombination Cloning Technology (Invitrogen, Carlsbad, CA, USA). pDONR221-human Surf4 or HMOX1 were then transferred into the destination vector pMXs-IP-GFP-frameB or pcDNA3.1-GFP-frameB, which incorporated the Gateway reading frame B cassette and N-terminal GFP sequence under the control of LTR or CMV promoters, respectively. pcDNA3-hInsulin-mRFP constructs used for expressing human insulin with C-terminal mRFP were provided by Dr. S. Torii (Gunma University, Maebashi, Gunma Japan). Full length human insulin (insulin-HaloTag)^41^ were subcloned into the pcDNA3-mRFP plasmid^42^. To construct NPY-Venus/pMRX-IRES-puro-DEST, the NPY-Venus cDNA was subcloned from pNPY-Venus-N1 vector (a kind gift from Dr. A. Miyawaki, RIKEN Brain Science Institute, Wako, Saitama, Japan)^55^ into Xho I/Not I site of the pENTR-3C vector. The vector was transferred into pMRX-IRES-puro-DEST vector (a kind gift from Dr. N. Fujita, Tokyo Institute of Technology, Tokyo, Japan)^56^ by Gateway system using LR clonase recombination reaction (Invitrogen).

### Antibodies

Rabbit anti-mRFP antibodies (1:1000 for immunoblotting) were generated as previously described^57^. Guinea pig anti-porcine insulin serum (1:1000 for immunostaining) was provided by H. Kobayashi^35^. Rat monoclonal anti-Sec12 (1:50 for immunostaining, 1:500 for immunoblotting) and anti-Sec23 (1:100 for immunostaining) and rabbit polyclonal anti-Sec16 (1:100 for immunostaining) antibodies were generated (and characterized) as described previously^58,59^. The following antibodies were purchased: mouse monoclonal anti-pan-actin (C4; Millipore, Tokyo, Japan, 1:3000 for immunoblotting), mouse monoclonal anti-GFP (3E6; Thermo Fisher Scientific, Tokyo, Japan, 1:1000 for immunoblotting), goat polyclonal anti-GFP conjugated to HRP (Fitzgerald Industries International, Flanders, NJ, USA, 1:1000 for immunoblotting), goat polyclonal anti-Surf4 (S-12; Santa Cruz Biotechnology, Santa Cruz, CA, USA, 1:1000 for immunoblotting), rabbit monoclonal anti-PDI (C81H6; Cell Signaling Technology, Danvers, MA, USA, 1:100 for immunostaining), rabbit polyclonal anti-ERGIC-53/p58 (E1031; Sigma-Aldrich, Tokyo, Japan, 1:500 for immunostaining), rabbit anti-rat chromogranin A (Y291; Yanaihara Institute Inc., Fujinomiya, Shizuoka, Japan, 1:1000 for immunostaining), mouse monoclonal anti-insulin (L6B10; Cell Signaling Technology, Danvers, MA, USA, 1:500 for immunoblotting), mouse monoclonal anti-proinsulin (3A1; ab8301, Abcam, Cambridge, MA, USA, 1:300 for immunostaining), normal mouse IgG (12-371; Merck Millipore, Tokyo, Japan), mouse monoclonal anti-GM130 (35/GM130; BD Biosciences, San Jose, CA, USA, 1:500 for immunostaining), and mouse monoclonal anti-TGN38 (2/TGN38; BD Biosciences, 1:200 for immunostaining).

### Transfection

For transient expression of GFP-hHMOX1 or hInsulin-mRFP, cells were transfected with each expression construct using Lipofectamine 3000 reagent (Invitrogen) according to the manufacturer’s instructions and incubated for 24 h.

### RNAi

INS-1 832/13 or INS-1 832/13 stably expressing GFP-hSurf4 cells were transfected twice with siRNA against the rat non-targeting control and rat Surf4 using Lipofectamine RNAiMAX reagent (Invitrogen) according to the manufacturer’s instructions. Briefly, cells (1.5 × 10^6^ mL^−1^) were suspended in RPMI 1640 medium without antibiotics, mixed with serum-free Opti-MEM medium (Invitrogen) containing Lipofectamine RNAiMAX reagent and siRNA (final concentration: 50 nM), plated in 6-well dishes, and incubated for 72 h. Cells were analyzed after the second transfection. RNAi oligonucleotides (ON-TARGET plus siRNA against rat Surf4: 5ʹ-CAGCGCGACUAUAUUGAUA-3ʹ, ON-TARGET plus siRNA against rat Surf4 #2: 5ʹ-GGACAAUCCCGGUCUAUAA-3ʹ) and control siRNA (ON-TARGET plus Non-targeting siRNA #3) were purchased from Dharmacon (Lafayette, CO, USA).

### Immunoblotting of mouse tissue lysates

Tissues from 8-week-old male C57BL/6 N mice were homogenized in ice-cold homogenate buffer (50 mM Tris-HCl at pH 7.4 containing 0.25 M sucrose and 1 mM EDTA) with a protease inhibitor cocktail using the mini homogenizer tube BioMasher II (Nippi, Tokyo, Japan). An equal amount of lysis buffer (50 mM Tris-HCl at pH 7.4 containing 250 mM NaCl, 1% Triton X-100, 0.1% SDS, 0.2% sodium deoxycholate, and 1 mM EDTA) was added to the homogenates. Tissue lysates were incubated on ice for 30 min and centrifuged at 15,000 rpm at 4 °C for 15 min. The protein concentration of the supernatants was quantified using a BCA assay kit (Pierce Chemical Company, Rockford, IL, USA).

### Immunoblotting and immunoprecipitation

To prepare whole-cell lysates, cells were washed in PBS and resuspended in a lysis buffer (50 mM Tris-HCl at pH 7.5 containing 150 mM NaCl, 1 mM EDTA, 1% Triton X-100 and supplemented with protease inhibitors) and incubated for 30 min at 4 °C, the cell lysates were centrifuged for 15 min at 15,000× *g* at 4 °C. Then, supernatants were mixed with Laemmli sample buffer, boiled for 60 min at 37 °C, and analyzed by immunoblotting with specific antibodies. For immunoprecipitation assays, cells were collected, washed with PBS, and resuspended in lysis buffer (50 mM Tris-HCl at pH 7.5 containing 150 mM NaCl, 1 mM EDTA, 0.1% NP-40 and supplemented with protease inhibitors). After incubation for 30 min at 4 °C, cell lysates were centrifuged for 15 min at 15,000× *g* at 4 °C. The supernatants were incubated with GFP-trap agarose beads (Chromotek, Planegg-Martinsried, Germany) for 15 min at 4 °C with gentle shaking. After incubation, beads were washed with lysis buffer without NP-40 five times, eluted with Laemmli sample buffer for 60 min at 37 °C, and subjected to immunoblotting.

To detect the interaction between GFP-hSurf4 and endogenous Surf4, the supernatants were incubated with anti-GFP (3E6) and anti-Surf4 (S-12) antibodies for 4 h at 4 °C, and the immune complexes were precipitated using protein G Sepharose beads (GE Healthcare, Piscataway, NJ, USA). To detect the interaction between GFP-hSurf4 and Sec12, the supernatants were incubated with Dynabeads^™^ Protein G (10003D; Thermo Fisher Scientific) coupled with anti-GFP (3E6) or normal mouse IgG for 60 min at room temperature. After incubation, the precipitated beads were washed and eluted with Laemmli sample buffer for 60 min at 37 °C, and the eluates were immunoblotted with specific antibodies.

### Immunofluorescence microscopy

INS-1 832/13 cells cultured on coverslips were fixed in 4% paraformaldehyde in PBS for 10 min, permeabilized, and blocked with 0.1% Triton X-100 in PBS containing 5% FBS and 0.3% bovine serum albumin (BSA) for 1 h. For Sec12 and Sec23 staining, cells were fixed in pre-cooled (−20 °C) methanol for 6 min, permeabilized and blocked with 0.1% Triton X-100 in PBS containing 5% BSA for 30 min, incubated with primary antibodies for 60 min, washed three times with PBS, and further incubated with Alexa Fluor 488-, 555-, or 594- conjugated secondary antibodies (1:1000 dilution; Invitrogen) for 60 min. The samples were washed five times, mounted using Slow Fade Diamond (Invitrogen) containing DAPI (157574; Molecular Probes, Eugene, OR, USA), and visualized using a Fluoview FV1200 confocal laser scanning microscope (Olympus Corporation, Tokyo, Japan) equipped with a 60× oil immersion objective lens (1.35 NA).

### Insulin secretion assay

INS-1 832/13 cells or INS-1 832/13 stably expressing GFP-hSurf4 cells were transfected twice with RNAi oligonucleotides as described above and treated with modified Krebs–Ringer buffer (KRB; containing 120 mM NaCl, 24 mM NaHCO_3_, 1 mM MgCl_2_, 2 mM CaCl_2_, and 15 mM HEPES at pH 7.4 and supplemented with 0.1% BSA and 2.8 mM glucose) for 2 h, followed by incubation with the same KRB buffer (low glucose) or that containing 25 mM glucose (high glucose) for 2 h. The amount of secreted insulin was measured using an AlphaLISA immunoassay kit (PerkinElmer, Waltham, MA, USA) according to the manufacturer’s instructions.

### Quantitative real-time PCR

INS-1 832/13 cells were incubated with KRB containing low (2.8 mM) or high (25 mM) glucose for 2 h at 37 °C, and total RNA was extracted with RNAiso plus (Takara, Tokyo, Japan) using a ReverTra Ace qPCR RT kit (Toyobo, Tokyo, Japan). cDNA was subjected to real-time PCR analysis with 1× SYBR Green PCR master mix (Applied Biosystems, Foster City, CA, USA) on Thermal Cycler ViiA7 System (Applied Biosystems). The respective primer sequences were as follows: for *Surf4*, forward, 5ʹ-GGACCCGAGAAGACCTCCTT-3ʹ and reverse, 5ʹ-GCACATCACTCAGAATTTCAATGG-3ʹ; for *ins-1*, forward, 5 -GCAAATGCTTCTAGGCGGAC −3ʹ and reverse, 5ʹ-AAGAAAGGGTGTAAAACGCAGC-3ʹ; for *β-actin*: forward, 5ʹ-GCAAATGCTTCTAGGCGGAC-3ʹ and reverse, 5ʹ-AAGAAAGGGTGTAAAACGCAGC-3ʹ.

### Quantification analysis

Colocalization analysis was performed using the Fiji software^60^. Each cell as a region of interest (ROI) was cropped, and Pearson’s correlation coefficient was determined using colocalization analysis with the Coloc 2 plugin. The number and size of puncta were quantified using the Analyze Particles algorithm in the Fiji software. The cell area was manually defined, and the punctate structures within the cytoplasmic area were filtered and counted. The number and size of the puncta were normalized to each cytoplasmic area. Western blot band intensities were quantified through densitometric scanning by using the chemiluminescence imaging system Fusion Solo S (Vilber-Lourmat, France) and normalized to loading control (β-actin). The mRNA levels were quantified by Quant Studio Real-Time PCR software v1.2 (Applied Biosystems) and normalized to β-actin.

### Statistics and reproducibility

Data were analyzed using GraphPad Prism 8 (GraphPad Software, Inc., San Diego, CA, USA) and are presented as bar graphs with the mean ± standard error or box and whisker plots generated using the Tukey method with the mean as “+”. The individual dots in bar graphs and box and whisker plots indicate the data points and the outliers, respectively. Statistical analysis were performed by using two-tailed unpaired Student’s *t*-tests or two-tailed unpaired *t*-test with Welch’s correction to evaluate significance and calculate *P* values. Two-tailed Mann–Whitney *U* tests were used for data with non-normal distribution. Statistical significance was set at *P* < 0.05. The sample size represented by n is shown in the figure legends. The experiments were not randomized and the investigators were not blinded. All experiments were performed at least three times with similar results.

### Reporting summary

Further information on research design is available in the Nature Research Reporting Summary linked to this article.

## Supplementary information

Supplementary Information

Description of Additional Supplementary Files

Supplementary Data 1

Reporting Summary

## Data Availability

All data supporting the results in this study are available from the corresponding author upon reasonable request. The original uncropped blot images can be found in Supplementary Fig. 7. Source data points behind the graphs can be found in the Supplementary Data.
