# Supplementary material for: Cargo receptor Surf4 regulates endoplasmic reticulum export of proinsulin in pancreatic β-cells
Source: Commun Biol. 2022 May 13;5:458. doi: 10.1038/s42003-022-03417-6 (PMC9106718; doi:10.1038/s42003-022-03417-6)
Supplement: Supplementary file 1 — Supplementary Information [file 42003_2022_3417_MOESM1_ESM.pdf]

Supplementary Information

Cargo receptor Surf4 regulates endoplasmic reticulum export of proinsulin in pancreatic β-cells

Keiko Saegusa[1], Kohichi Matsunaga[2], Miharu Maeda[3], Kota Saito[3], Tetsuro Izumi[2], and Ken Sato[1,*]

[1]Laboratory of Molecular Traffic, Institute for Molecular and Cellular Regulation, Gunma University, Maebashi, Gunma 371-8512, Japan

[2]Laboratory of Molecular Endocrinology and Metabolism, Department of Molecular Medicine, Institute for Molecular and Cellular Regulation, Gunma University, Maebashi, Gunma 371-8512, Japan

[3]Department of Biological Informatics and Experimental Therapeutics, Graduate School of Medicine, Akita University, Akita, 010-8543, Japan

*Correspondence: Ken Sato, PhD

Laboratory of Molecular Traffic

Institute for Molecular and Cellular Regulation, Gunma University

Maebashi, Gunma 371-8512, Japan

Phone: +81-27-220-8840

Fax: +81-27-220-8844

Email: sato-ken@gunma-u.ac.jp

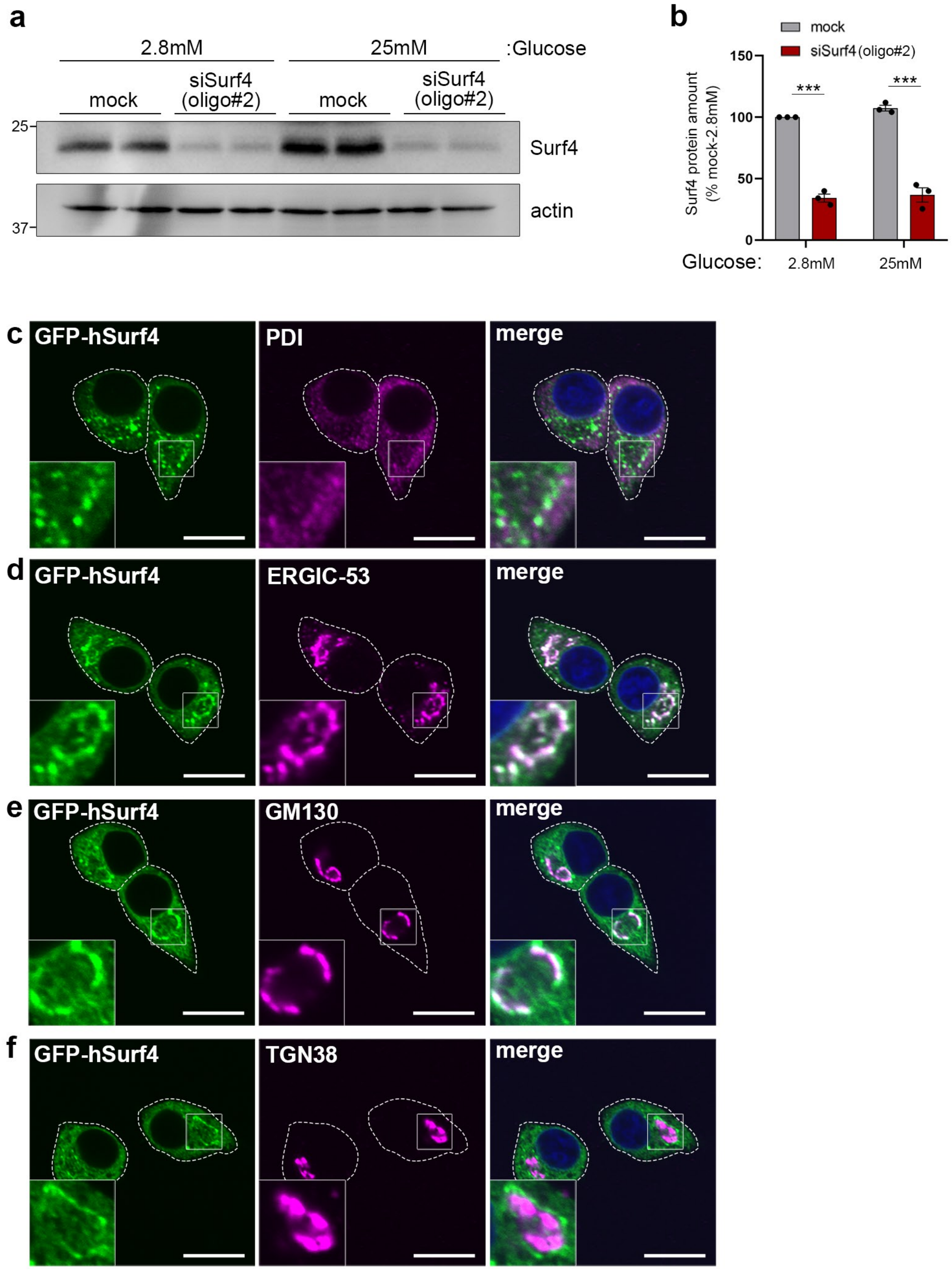

Supplementary Figure 1. Subcellular localization of GFP-hSurf4 in INS-1 832/13 cells.

a INS-1 832/13 cells were transfected with the mock or Surf4 siRNA (siSurf4 oligo#2) and incubated in KRB containing low (2.8 mM) or high (25 mM) glucose concentrations. After incubation for 2 h, whole-cell lysates were subjected to immunoblotting analysis using anti-Surf4 and anti-β-actin antibodies. **b** Quantification of Surf4 protein levels (n = 3) of INS-1 832/13 cells treated with the mock or Surf4 siRNA (siSurf4 oligo#2) following low- or high-glucose stimulation. Data are presented as the mean ± SEM and analyzed by two-tailed unpaired Student's t-tests, ***$p < 0.01$. **c-f** INS-1 832/13 cells stably expressing GFP-tagged human Surf4 was stained with anti-PDI (ER), ERGIC-53 (ERGIC), GM130 (cis-Golgi), and TGN38 (trans-Golgi network) antibodies. GFP-hSurf4 localized to the ER reticular structures and some punctate structures that were adjacent to ERGIC-53 and GM130. Dotted lines indicate cell outlines. Scale bars: 10 μm.

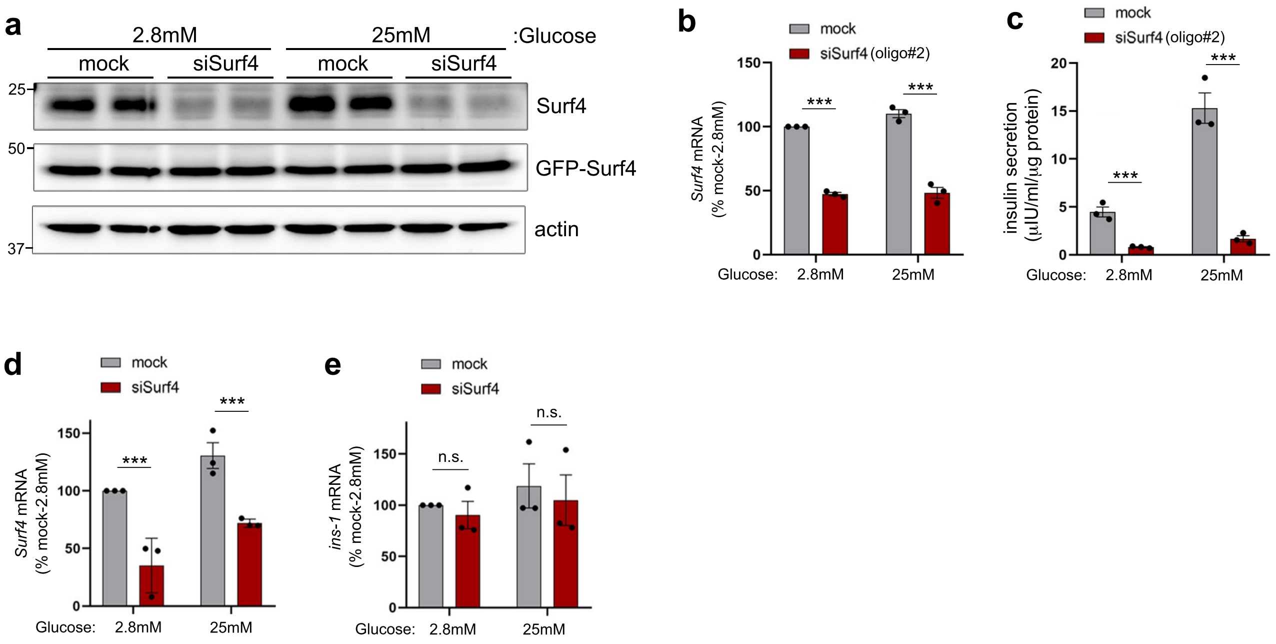

Supplementary Figure 2. The impairment of insulin secretion resulted from the loss of Surf4.

a INS-1 832/13 cells stably expressing GFP-hSurf4, whose sequence is resistant to siRNA against rat Surf4, were transfected with the mock or Surf4 siRNA (siSurf4) and incubated in KRB containing low (2.8 mM) or high (25 mM) glucose concentrations. After incubation for 2 h, whole-cell lysates were subjected to immunoblotting analysis using anti-Surf4, anti-GFP and anti-β-actin antibodies. **b, c** Quantification of Surf4 mRNA levels (b; $n = 3$) and the amount of secreted insulin (c; $n = 3$) of INS-1 832/13 cells treated with the mock or Surf4 siRNA(siSurf4 oligo#2) following low- or high-glucose stimulation. Data are presented as the mean ± SEM and analyzed by two-tailed unpaired Student's t-tests, ***$p <$ 0.01. **d, e** Quantification of Surf4 mRNA (d; $n = 3$) and ins-1 mRNA (e; $n = 3$) of INS-1 832/13 cells treated with the mock or Surf4 siRNA following low- or high-glucose stimulation. Data are presented as the mean ± SEM and analyzed by two-tailed unpaired Student's t-tests, ***$p < 0.01$; n.s., not significant.

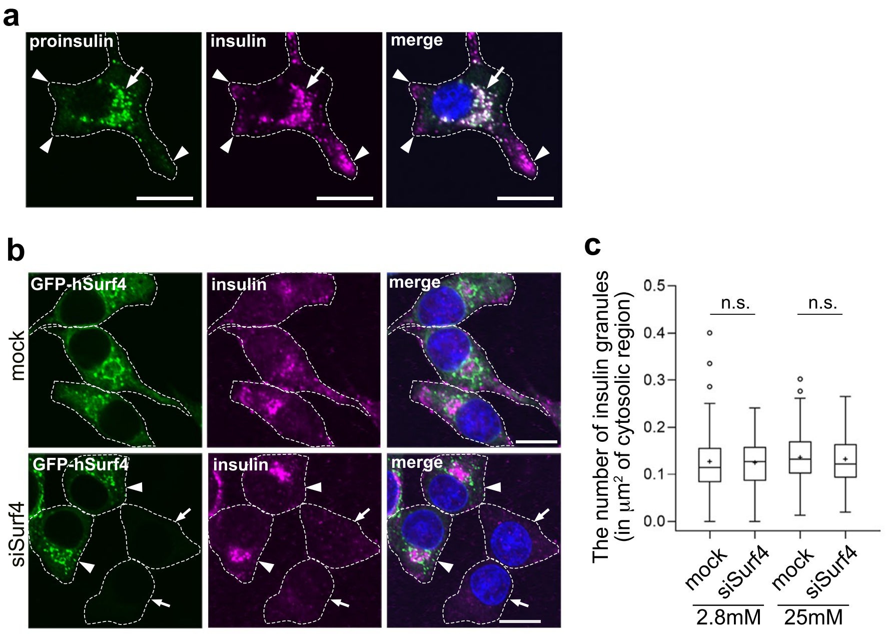

Supplementary Figure 3. Loss of Surf4 caused the reduction of insulin-containing secretory granules.

a INS-1 832/13 cells were fixed and stained with an anti-proinsulin antibody and an anti-insulin antibody that recognizes both proinsulin and mature insulin. Proinsulin-positive granules represent immature insulin granules (arrows). The secretory granule, which were stained with the anti-insulin antibody but not with the anti-proinsulin antibody, represent mature insulin granules (arrowheads). **b** Subcellular localization of insulin granules in INS-1 832/13 cells stably expressing a siRNA-resistant GFP-hSurf4 treated with the mock or Surf4 siRNA. Surf4 siRNA decreased the number of insulin-positive granules in INS-1 832/13 cells that did not express GFP-hSurf4 (arrows) but not in INS-1 832/13 cells expressing GFP-hSurf4 (arrowheads). **c** Quantification of the number of insulin granules in cells expressing GFP-hSurf4 ($n = 65$, mock- and siSurf4-transfected cells at low-glucose conditions; $n = 66$, mock-transfected cells at high-glucose conditions; $n = 69$, siSurf4-transfected cells at high-glucose conditions). Data are presented as the box and whisker plots and analyzed by two-tailed unpaired Student's *t*-tests, n.s., not significant. Dotted lines indicate cell outlines. Scale bars: 10 µm.

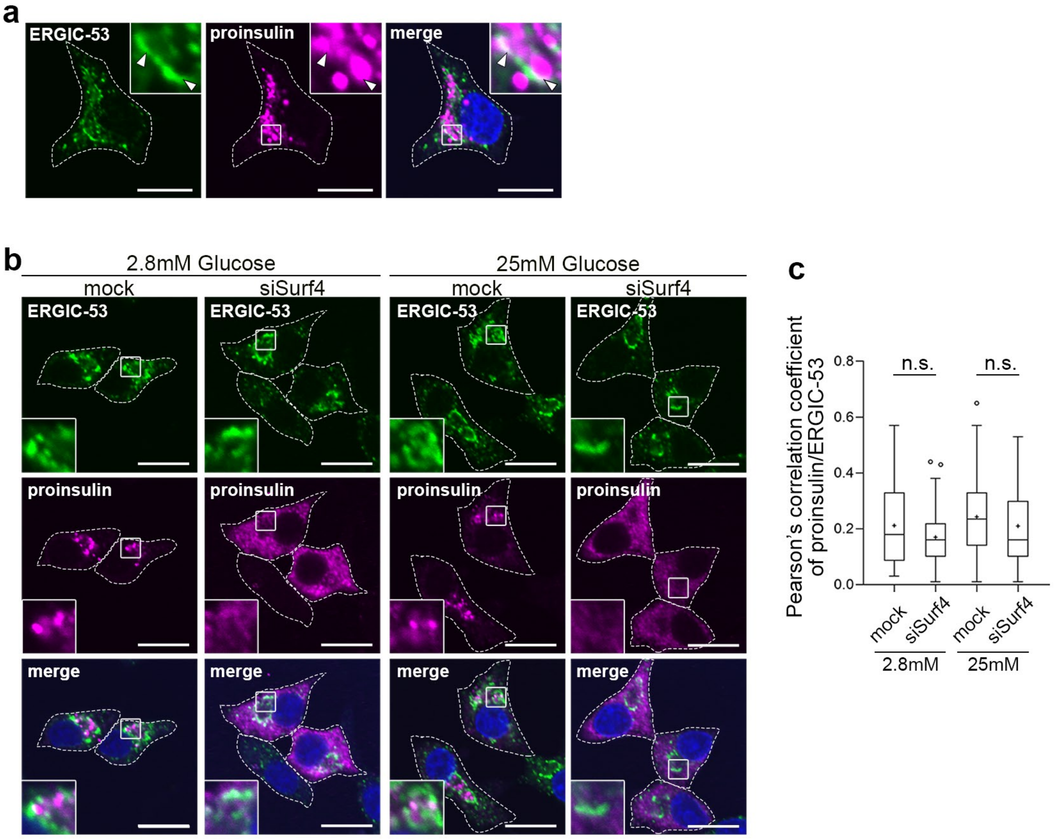

Supplementary Figure 4. Loss of Surf4 caused the accumulation of proinsulin in the ER but not in the ERGIC.

a A part of proinsulin localized to the ERGIC at a steady state. INS-1 832/13 cells were fixed and stained with anti-ERGIC-53 and anti-proinsulin antibodies. **b** Loss of Surf4 did not increase the accumulation of proinsulin in the ERGIC. INS-1832/13 cells were transfected with the mock or Surf4 siRNA and incubated in KRB containing low (2.8 mM) and high (25 mM) glucose concentrations for 2 h. Cells were fixed and stained with anti-ERGIC-53 and anti-proinsulin antibodies. **c** Quantification of colocalized proinsulin and ERGIC-53 as calculated by Pearson's correlation coefficient (n = 45, mock-transfected cells at low-glucose conditions; n = 47, siSurf4- transfected cells at low-glucose conditions; n = 46, mock-transfected cells at high-glucose conditions; n = 60, siSurf4-transfected cells at high-glucose conditions). Data are presented as the box and whisker plots and analyzed by two-tailed unpaired Student's t-tests, n.s., not significant. Dotted lines indicate cell outlines. Scale bars: 10 μm.

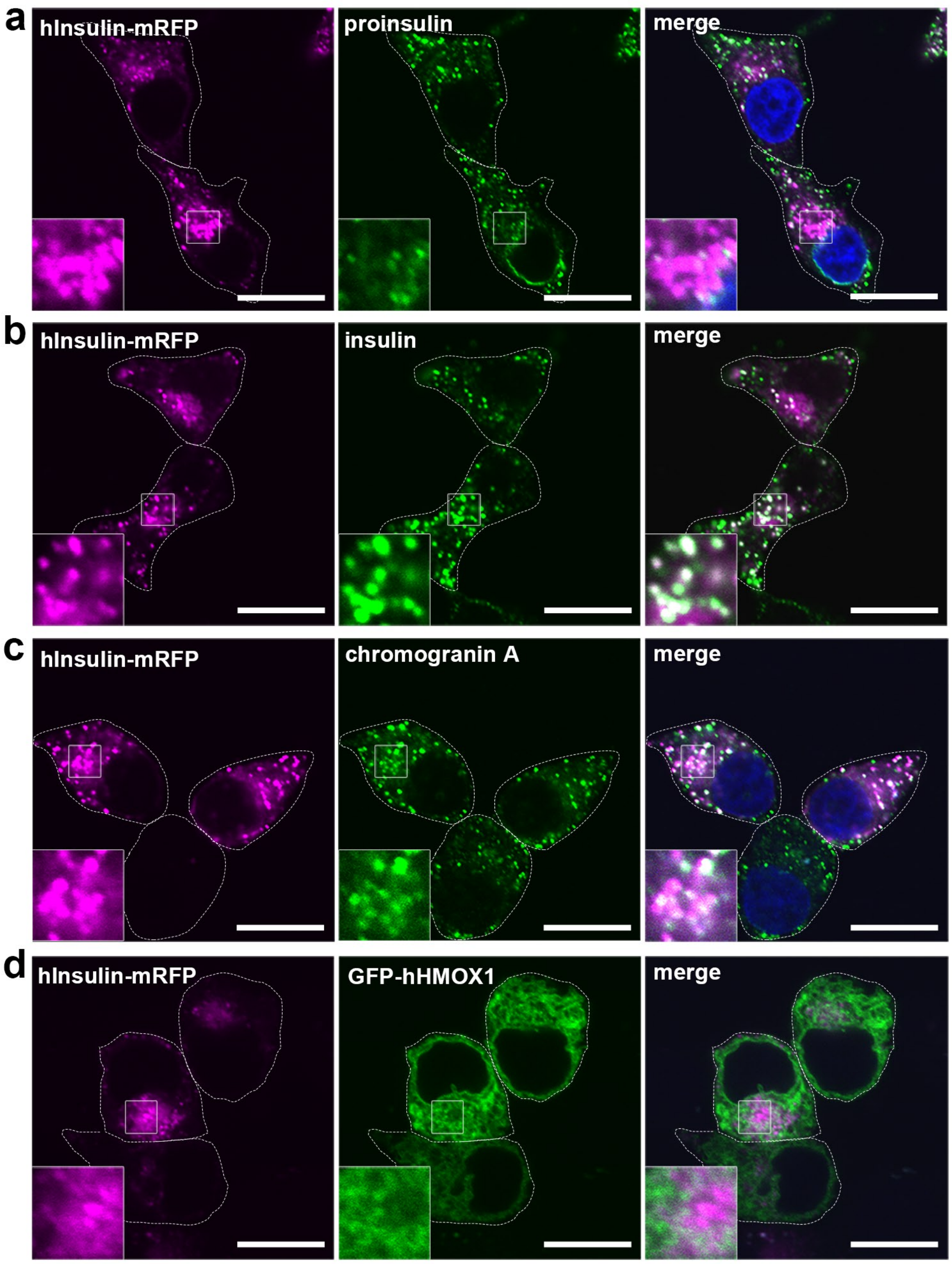

Supplementary Figure 5. hInsulin-mRFP was properly generated and transported into the secretory granules.

a-c INS-1 832/13 cells were transiently transfected with human insulin with a C-terminal mRFP tag (hInsulin-mRFP), fixed, and stained with anti-proinsulin, anti-insulin and anti-chromogranin A antibodies. hInsulin-mRFP localized to many granular structures that overlapped with proinsulin-, insulin-, and chromogranin A-positive granules. **d** INS-1 832/13 cells were transiently transfected with GFP-hHMOX1 and hInsulin-mRFP. GFP-hHMOX1 showed ER-like reticular structures. Dotted lines indicate cell outlines. Scale bars: 10 μm.

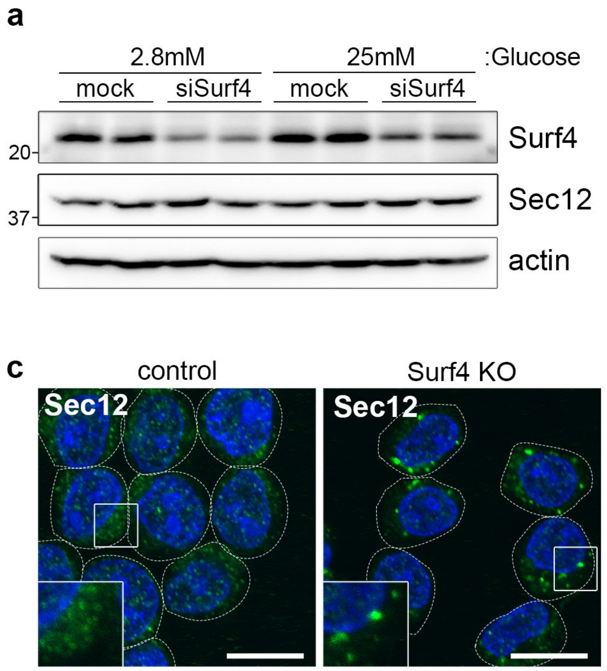

Supplementary Figure 6. Loss of Surf4 affected the subcellular distribution of Sec12 but not its protein level.

a INS-1832/13 cells were transfected with the mock or Surf4 siRNA and incubated in KRB containing low (2.8 mM) or high (25 mM) glucose concentrations for 2 h. Whole-cell lysates were subjected to immunoblotting analysis using anti-Surf4, anti-Sec12, and anti-β-actin antibodies. **b** Quantification of the relative level of Sec12 proteins in (a) by densitometric scanning of band intensities. Data are presented as the mean ± SEM and analyzed by unpaired Student's *t*-tests, n.s., not significant. **c** Control and Surf4-deleted HAP1 cells were fixed and stained with an anti-Sec12 antibody. Loss of Surf4 caused the redistribution of Sec12 to larger membrane structures, presumably in the ER. Dotted lines indicate cell outlines. Scale bars: 10 μm.

Figure 1a

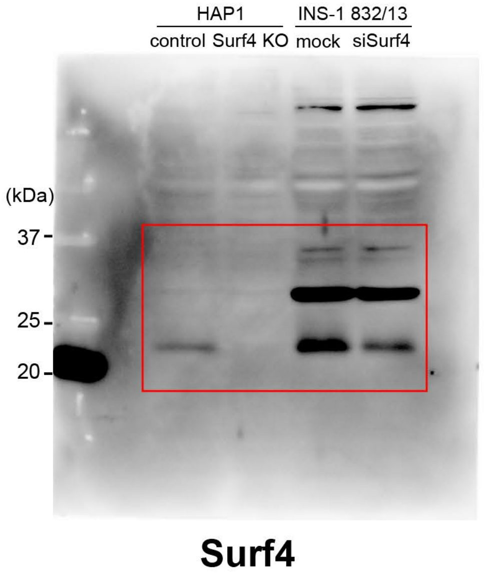

Figure 1b

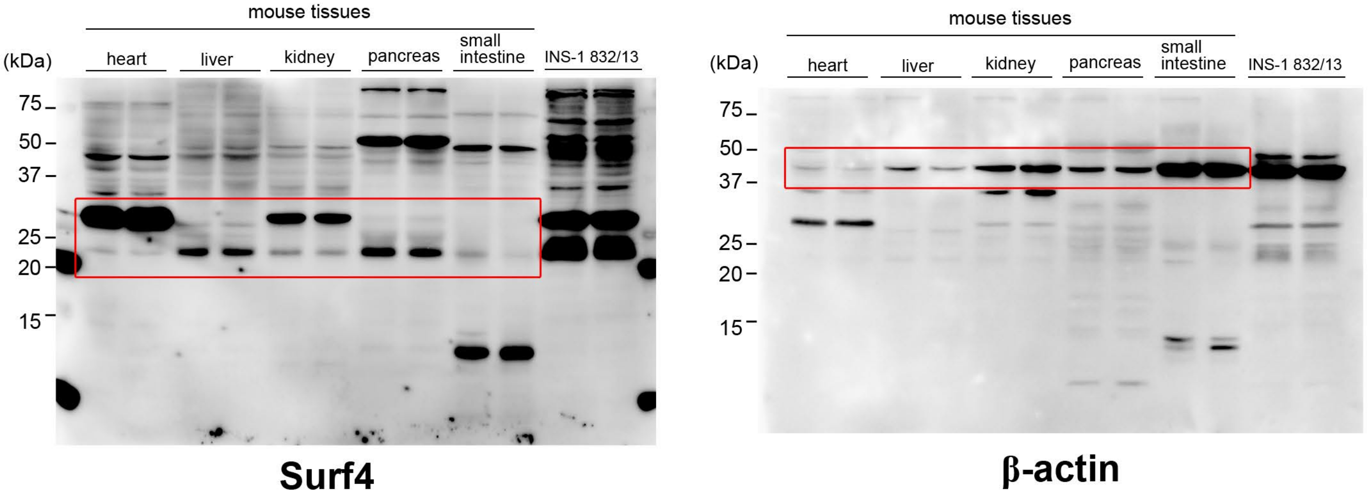

Figure 1c

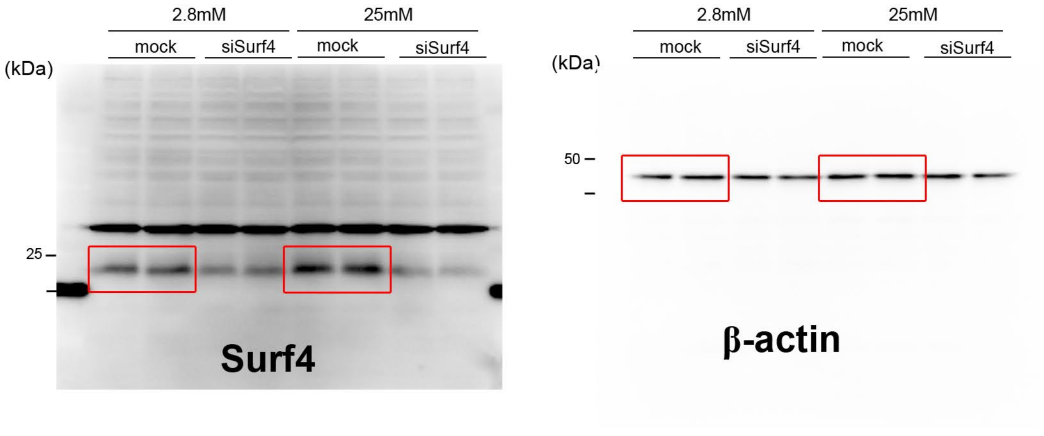

Figure 2d

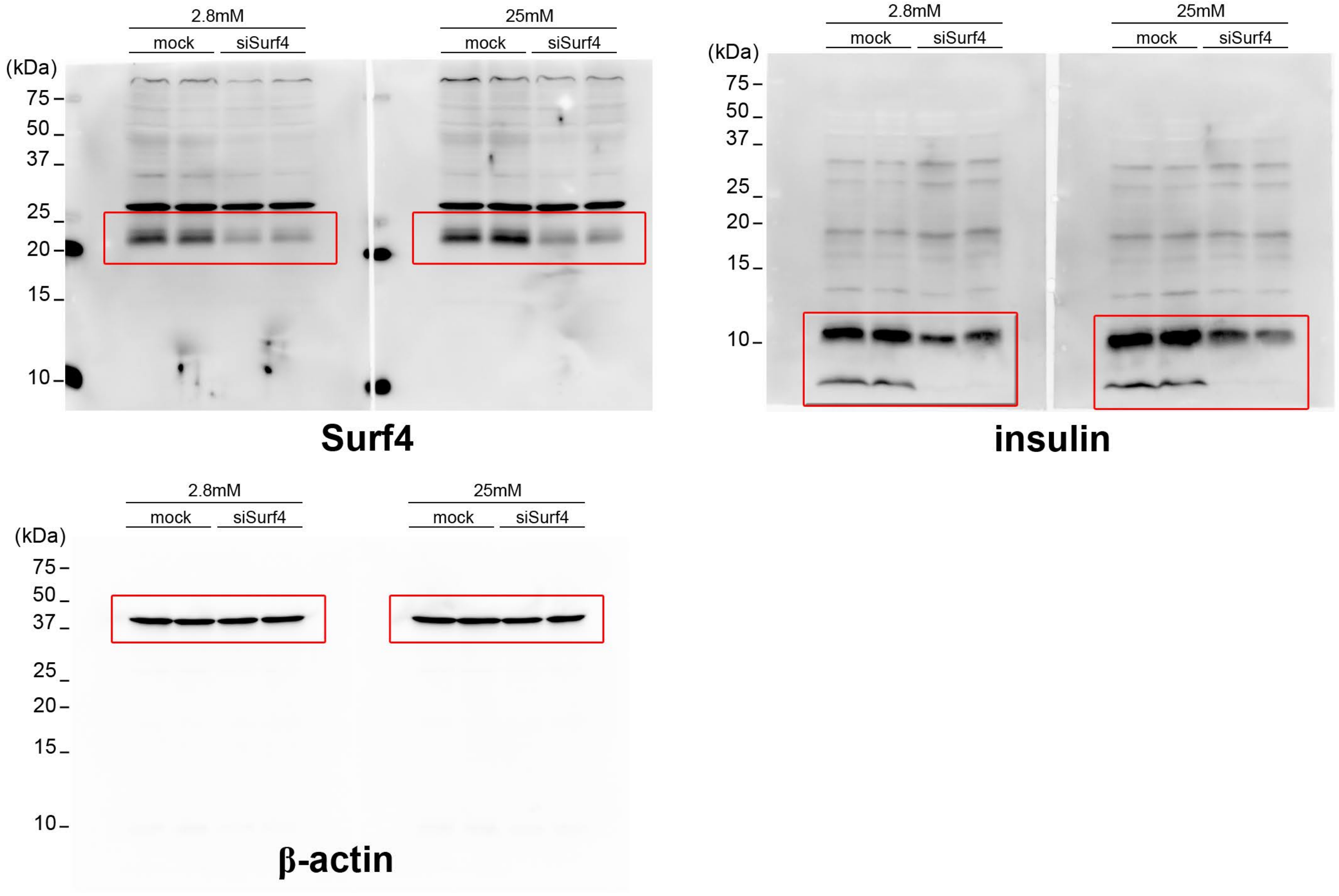

Figure 5a

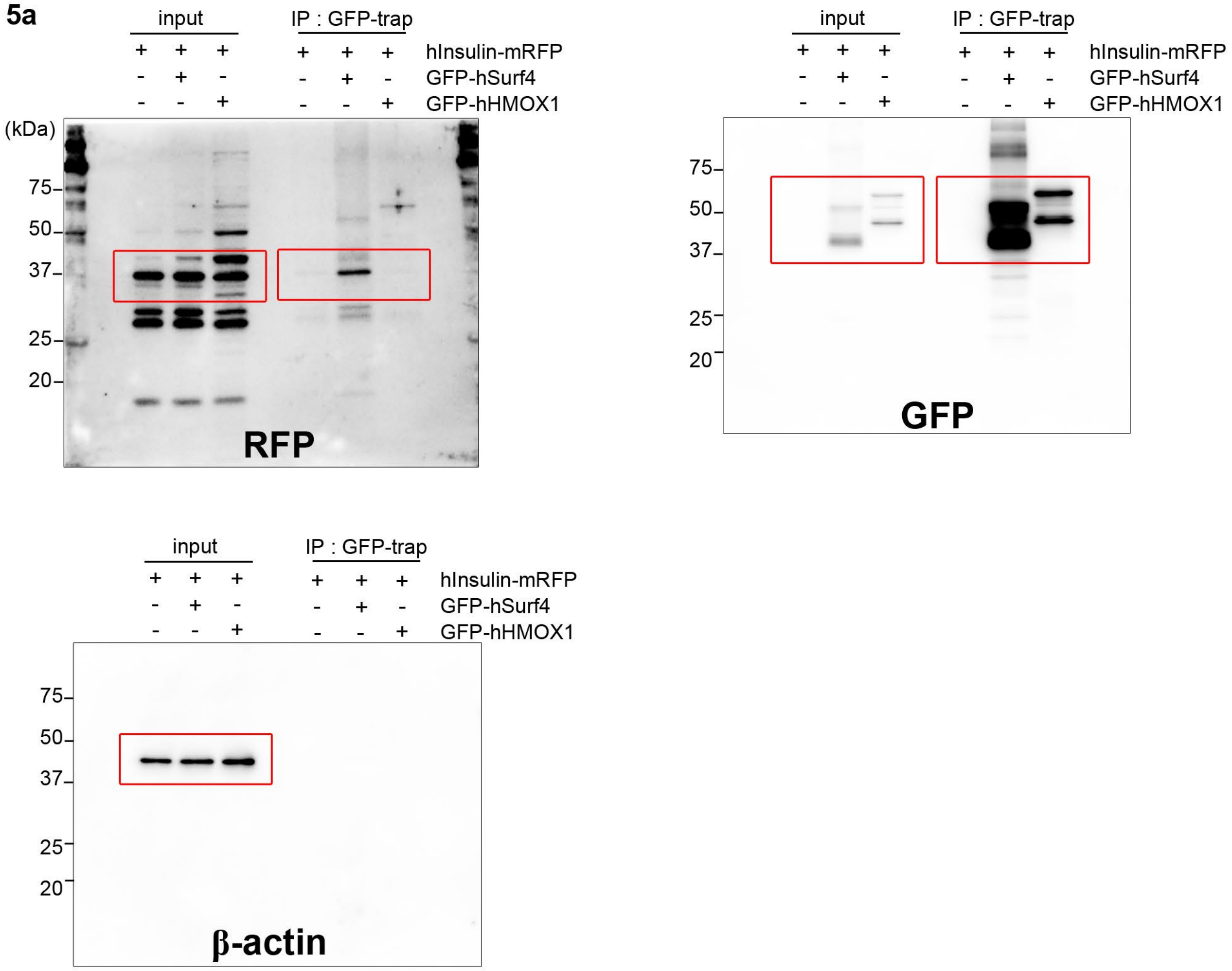

Figure 5b

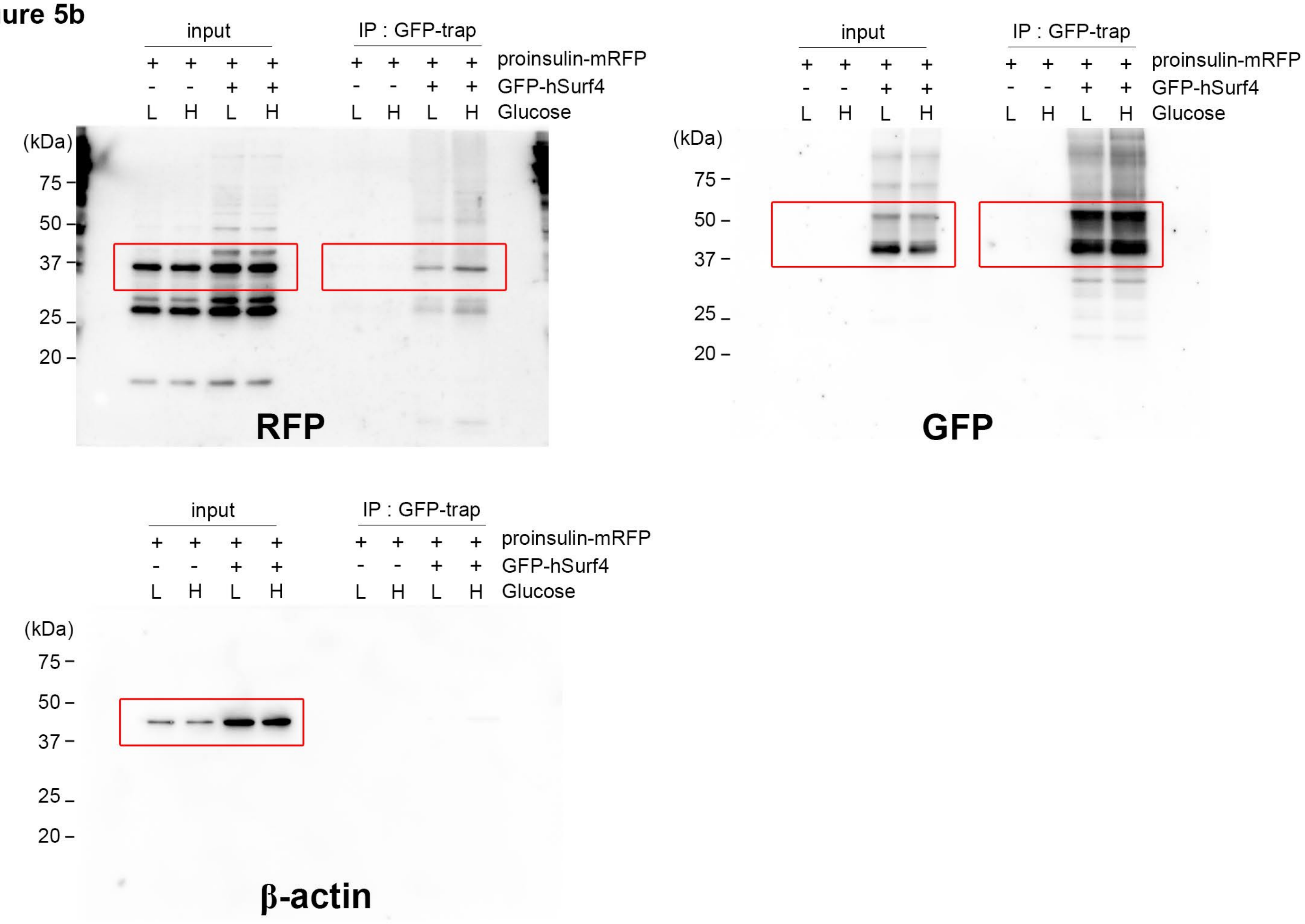

Figure 5d

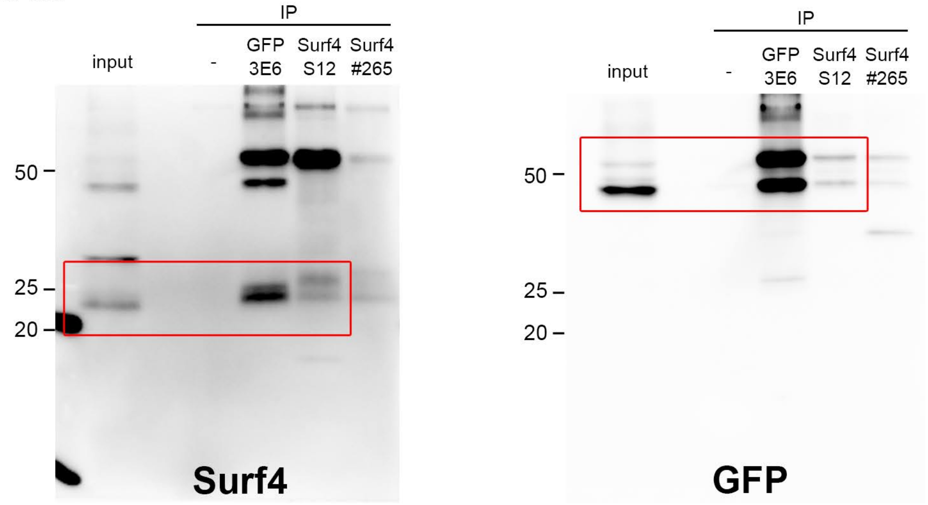

Figure 7e

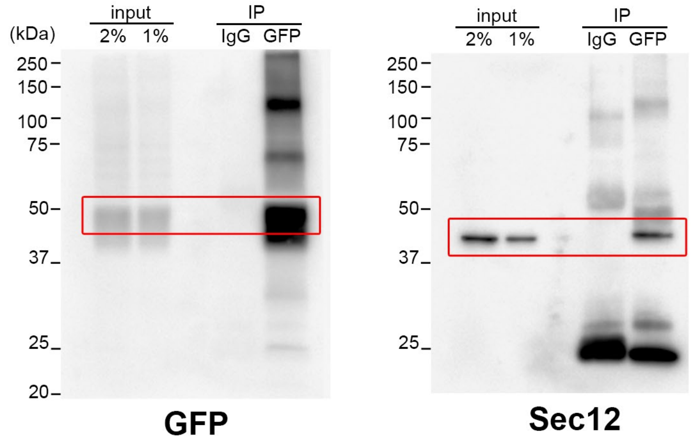

Figure S1a

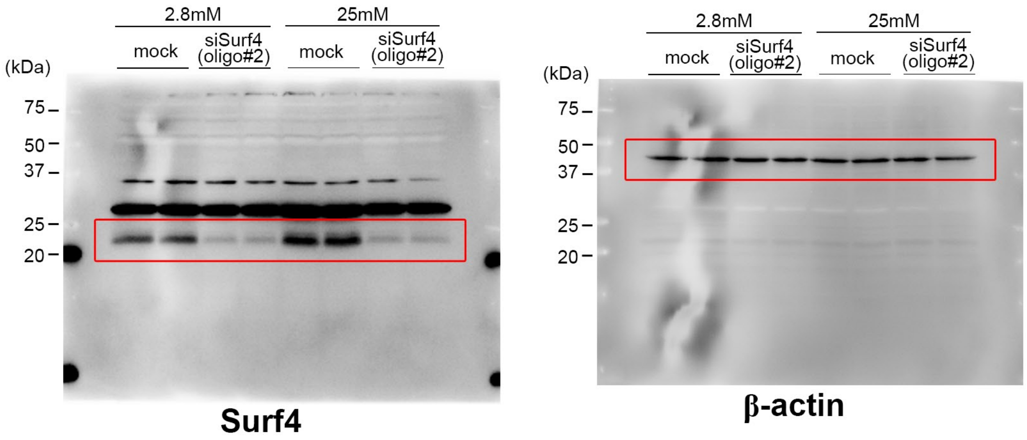

Figure S2a

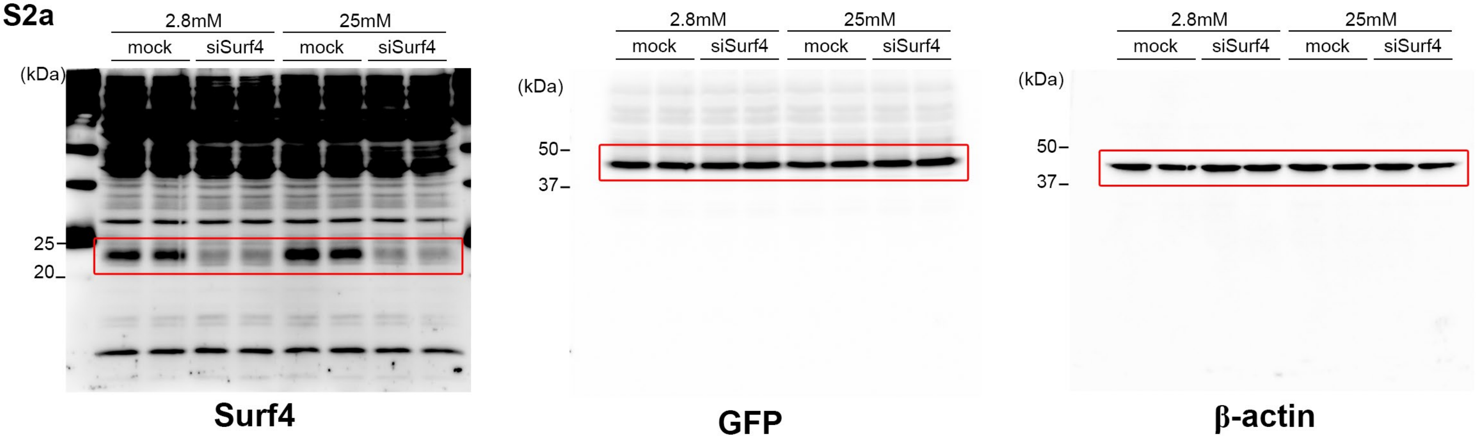

Figure S6a

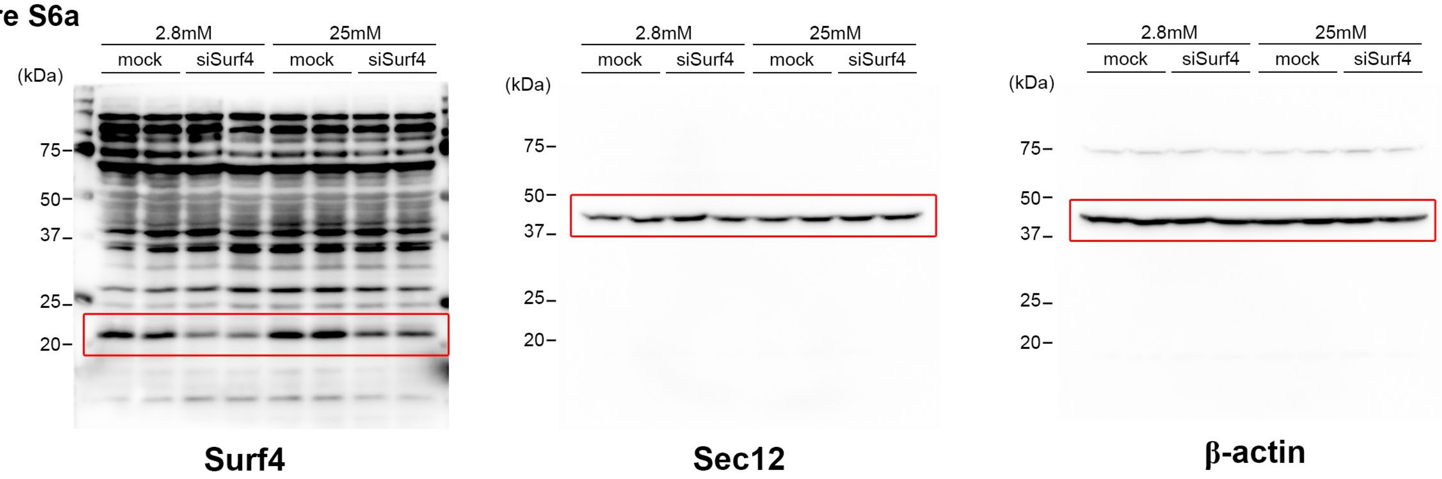

Supplementary Figure 7. Uncropped Western blots.
The lanes represented in the main figures are marked with a red rectangles.
