# Supplementary material for: Cargo receptor Surf4 regulates endoplasmic reticulum export of proinsulin in pancreatic β-cells
Source: Commun Biol. 2022 May 13;5:458. doi: 10.1038/s42003-022-03417-6 (PMC9106718; doi:10.1038/s42003-022-03417-6)
Supplement: Supplementary file 2 — Description of Additional Supplementary Files [file 42003_2022_3417_MOESM2_ESM.pdf]

Description of Additional Supplementary Files

File name: Supplementary Data
Description: Source data points behind the graphs.
